# Coacervate‐Mediated Lysosome‐Targeting Antibody Delivery for Protein Degradation

**DOI:** 10.1002/advs.202520441

**Published:** 2026-02-25

**Authors:** Dingdong Yuan, Yishu Bao, Zhiyi Xu, Zhong Zheng, Yongxu Han, Kai Cheng, Yuan‐Di Zhao, Jiang Xia

**Affiliations:** ^1^ Department of Chemistry The Chinese University of Hong Kong Shatin Hong Kong SAR China; ^2^ Britton Chance Center for Biomedical Photonics at Wuhan National Laboratory for Optoelectronics‐Hubei Bioinformatics and Molecular Imaging Key Laboratory Department of Biomedical Engineering College of Life Science and Technology Huazhong University of Science and Technology Wuhan Hubei China

**Keywords:** intracellular delivery, lysosome‐targeting delivery, peptide coacervates, phase separation, targeted protein degradation

## Abstract

Selective recognition of cancer‐associated proteins (CAPs) by antibodies, followed by their delivery into the intracellular organelle, the lysosome, results in targeted degradation of CAPs and suppresses the growth of cancer cells. Translocating the antibody—CAP complex across the plasma membrane is, however, nontrivial. Phase‐separating molecules are known to form membrane‐translocating coacervates that can encapsulate proteins and transport them into the cytoplasm. Nevertheless, these coacervates generally lack the ability to guide the cargo to the lysosome. Here, we seal this gap and develop lysosome‐targeting coacervates by tailoring a tetrapeptide into a phase‐separating, coacervate‐forming peptide. In the aqueous solution, the peptide derivative forms microdroplets, or coacervates, through liquid‐liquid phase separation (LLPS), which spontaneously enter cells and colocalize with the lysosome; hence, these coacervates are referred to as Lysosome‐Sorting Peptide Coacervates or **LSP‐Coa**. We show that **LSP‐Coa** can encapsulate proteins, facilitate the translocation of antibody–CAP complexes to the lysosome, and enable the degradation of membrane‐bound CAPs — a mechanism we call Coacervate‐mediated Lysosome‐targeting Protein Degradation, or **CoaLPD**. Using the **CoaLPD** technology, we successfully degraded HER2 and EGFR in cancer cells and in tumor‐bearing mice, showcasing its potential use as an anticancer treatment. The **LSP‐Coa** system also increases the efficacy of PROTAC degradation through enhanced lysosomal uptake. Taken together, we present the design of lysosomal‐targeting coacervates and demonstrate their use as vehicles for lysosome‐specific antibody delivery and for the selective degradation of CAPs, thereby validating the **CoaLPD** strategy as a potential anticancer treatment.

## Graphical abstract

Targeting coacervates to the lysosome in the cytoplasm is a challenge. Here, we convert a tetrapeptide into lysosome‐targeting, membrane‐translocating coacervates. The lysosome‐sorting peptide coacervates facilitate coacervate‐mediated delivery of antibody antigen complexes and a PROTAC compound to the lysosome for the targeted degradation of cancer‐associated proteins.

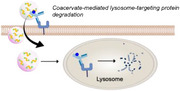

## 1 Introduction

Traditional drug discovery relies on navigating the chemical space of small‐molecule compounds to identify inhibitors for disease‐causing proteins. However, not all disease‐causing proteins are amenable to site‐selective inhibition or blockage. For those protein targets traditionally considered undruggable, researchers have succeeded in modulating their activity by targeting their mRNA or DNA with genetic tools. A recently emerged strategy of knocking down the protein of interest is directing it to cellular machinery, such as the proteasome or lysosome, achieving targeted protein degradation (TPD). Since the introduction of the proteolysis‐targeting chimera (PROTAC) technology by Sakamoto, Crews, Deshaies, and their colleagues [1, 2, 3, 4, 5], to date, more than 20 PROTACs have entered various stages of clinical studies for treating cancers, with some in Phase III trials. The groundbreaking innovation of PROTAC thus leads to the validation of TPD as a promising cancer treatment strategy.

Notwithstanding, many cancer‐associated proteins (CAPs) are membrane‐bound and thus beyond the realm of PROTAC degraders. To degrade membrane‐bound proteins, Bertozzi and coworkers pioneered the development of the lysosome‐targeting chimera (LYTAC), which consists of an antibody targeting the CAP and a glycopeptide that binds to a lysosomal targeting receptor (LTR) located on the cell surface, such as the mannose‐6‐phosphate receptor [6]. The interaction between the LTR and the LYTAC–membrane protein complex facilitates their entry into the cells and their further intracellular translocation to the lysosome, which incurs degradation. The invention of the LYTAC technology stimulated studies on antibody‐guided lysosome‐dependent TPD. Since then, various LTRs, including asialoglycoprotein receptors (ASGPR) [7], glucose transporter 1 (Glut1) [8], the membrane‐bound E3 ligase Ring finger protein 43 (RNF43) [9], the C‐X‐C chemokine receptor type 7 (CXCR‐7) [10], the transferrin receptor (TfR) [11], and the low‐density lipoprotein receptor‐related protein 1 (LRP1) [12], have been explored to internalize antibody–membrane protein complexes for lysosomal degradation. However, the degradation efficacy of these receptor‐dependent strategies heavily relies on the expression levels of LTRs, which may vary among different cells [13, 14, 15, 16, 17].

Receptor‐independent lysosome‐targeting degradation strategies have also been reported. For example, Chen and coworkers generated an engineered nanobody chimera (GlueTAC) that contains a proximity‐based covalent binding site between the antigen and the nanobody, a cell‐penetrating peptide (CPP) that facilitates the entry of the entire complex into cells, and a lysosome‐sorting sequence that enhances the lysosomal trafficking of GlueTAC to achieve antigen degradation [18, 19]. Researchers also use the autophagy pathway to degrade membrane proteins through a receptor‐independent strategy. For instance, an antibody‐peptide conjugate, Ab‐CMA, targets chaperone‐mediated autophagy (CMA), leading to TPD [20]. Compared to receptor‐dependent methods, the effectiveness of LTR‐independent methods does not rely on the receptor's expression level. Nevertheless, for all antibody‐dependent lysosome‐targeting TPDs, two steps are critical: the internalization of the antibody–membrane protein complexes and their intracellular translocation to the lysosome. Either LTRs or cell‐penetrating peptides are required to deliver antibody–membrane protein complexes across the plasma membrane into cells and ensure the targeted translocation of the complexes to the lysosome.

On another note, we recently discovered that molecular coacervates formed through liquid‐liquid phase separation (LLPS) could spontaneously deliver antibodies into cells, a new strategy in cross‐membrane translocation of biopharmaceuticals. Molecules containing multiple weakly interacting motifs (for example, hydrophobic groups) and a hydrophilic linker, meeting the “stick‐and‐spacer model,” are prone to undergo LLPS and form microdroplets or coacervates [21]. The coacervate formation grants small molecule compounds properties that a single molecule does not have [22]. Remarkably, as an aggregation state, the coacervates can encapsulate different biopharmaceuticals and deliver them into liposomes and cells [23, 24]. Starting from coacervating polymers [25, 26] to biomolecules and phase‐separating peptides, including the mixture of the histone and DNA [27, 28], L17E trimer [29], HBpep‐SR [30, 31, 32, 33, 34, 35], and others [36, 37, 38], coacervate‐mediated delivery has been realized by a variety of peptides and proteins. In addition, we report that low‐molecular‐weight small molecules can form coacervates, convoy proteins into cells, and release the cargo upon different chemical reactions inside the cell. For example, we designed a photo‐responsive, phase‐separating fluorescent molecule (PPFM) with a molecular weight of 666.6 Daltons based on pyrene that can undergo LLPS in the aqueous solution, carry proteins into cells, and release the cargo to the cytosol upon photo illumination [39]. We also designed a triphenylphosphine‐based compound that can deliver proteins into cells and respond to an azide compound for cytosolic release [40]. Utilizing these molecular coacervates, we successfully delivered both an antibody and an E3 ubiquitin ligase TRIM21 into cancer cells, realizing selective TPD of CAPs in cancer cells and significant inhibition of tumor sizes in animals.

Despite the vigorous growth of the coacervate‐mediated delivery field, to our knowledge, none of the coacervates have achieved organelle‐targeted delivery. We envision that redesigning the known lysosome‐specific peptides to phase‐separated peptide coacervates while maintaining their lysosome‐sorting capability will develop lysosome‐targeting coacervates to achieve both transmembrane delivery and lysosome‐specific targeting at the same time. We recently demonstrated that incorporating the Fmoc group and the hydrophobic 3,4‐dihydroxyphenylalanine at the two termini of a peptide allows the development of phase‐separating, coacervate‐forming peptides [41]. Therefore, here, we design and synthesize peptide coacervates with lysosome‐targeting features and showcase the degradation of membrane‐bound CAPs both in cancer cells and in vivo.

## 2 Results and Discussion

### 2.1 Design and Synthesis of the LSP‐Coa

**FIGURE 1:**
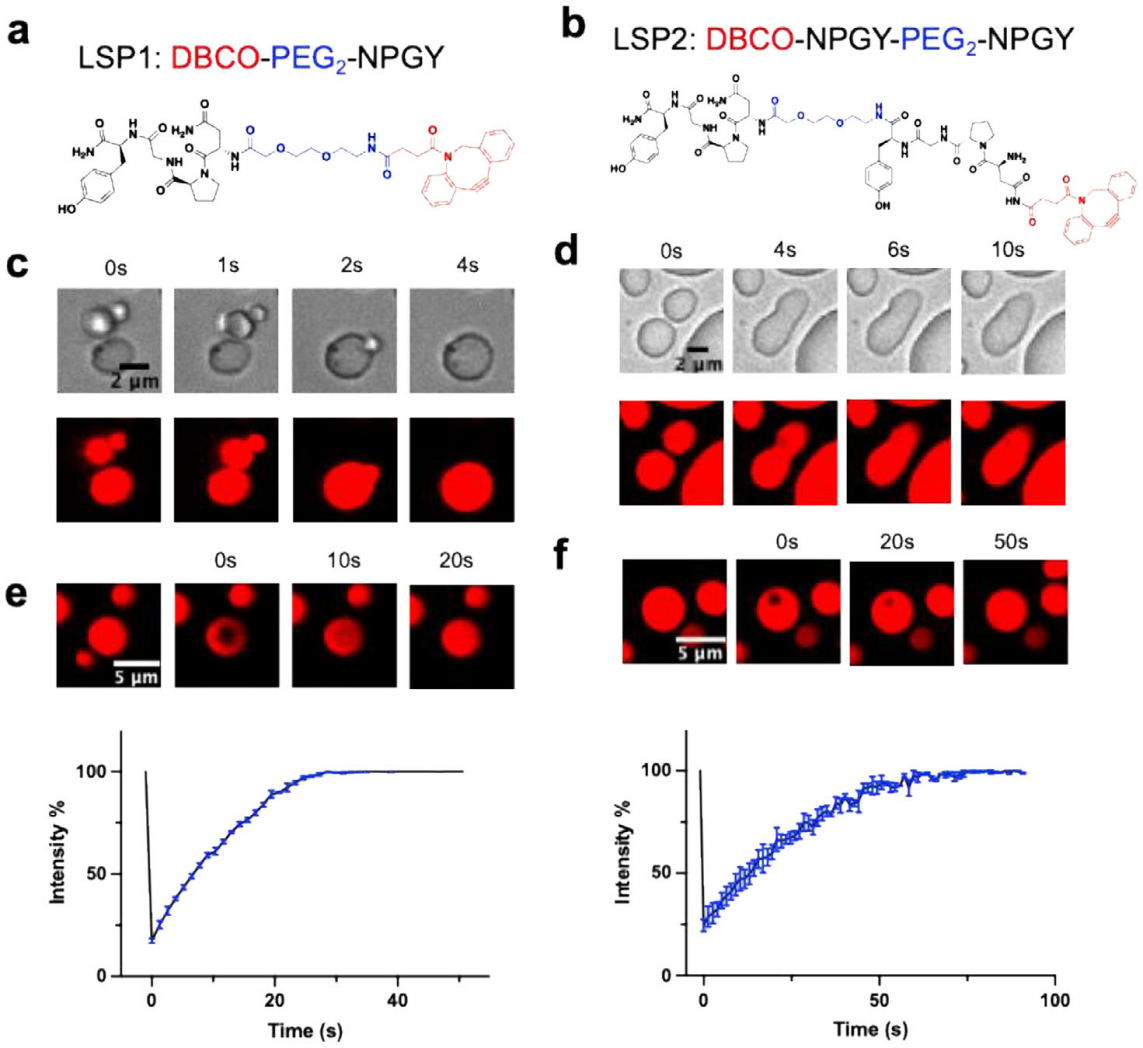
Design and characterization of phase‐separating peptides, LSP1 and LSP2. (a,b) Chemical structures of LSP1 and LSP2. (c,d) Fluorescent microscopic images showing the fusion of LSP1 droplets and LSP2 droplets. (e,f) Fluorescence recovery after photobleaching (FRAP) analysis showing the fluorescence recovery of LSP1 coacervates and LSP2 coacervates after photobleaching. Red: Nile Red, 0.1 mg/mL; peptides, 3 mg/mL.

To convert a soluble peptide into a phase‐separating peptide, we reason that we can redesign it according to the “sticker‐and‐spacer” rule while preserving its target‐binding capability [30]. Based on the lysosome‐specific tetrapeptide Asn‐Pro‐Gly‐Tyr (NPGY), we modified its N‐terminus by adding a flexible, hydrophilic PEG linker and a hydrophobic moiety, dibenzocyclooctyne (DBCO), as a sticker. After screening a library of peptide derivatives, we identified two peptides, LSP1 (DBCO‐PEG_2_‐NPGY) and LSP2 (DBCO‐NPGY‐PEG_2_‐NPGY), that formed microdroplets in the phosphate‐buffered saline (PBS) at pH 7.4 (Table S1). Briefly, peptides were first dissolved in dimethyl sulfoxide (DMSO) as a stock solution (100 mg/mL). When the stock solution was diluted into PBS, microdroplets formed. Under a fluorescent confocal microscope, the microdroplets labeled with Nile Red could spontaneously fuse into larger droplets (Figure 1a–d). After photobleaching a portion of the droplet, the fluorescence of the photobleached region could quickly recover within one minute to 80 % of the signal intensity before bleaching (Figure 1e,f). According to the kinetics of fluorescence recovery, the diffusion coefficients (D) of the coacervates of LSP1 and LSP2 peptides were measured to be about 2.84 × 10^−2^ and 4.33 × 10^−3^ µm^2^/s, respectively [42]. These two characteristics indicated that these microdroplets had liquid‐like properties.

Next, we examined the cytotoxicity of the peptide coacervates to cancer cells. LSP1 and LSP2 were added to SK‐BR‐3 cells at different concentrations, respectively. After 12 h of incubation, cell viability was assessed using the CCK‐8 assay (Figure S1). Interestingly, peptide LSP1 showed rather high cytotoxicity, whereas peptide LSP2 had no significant cytotoxicity at a concentration of 1 mg/mL. Because both peptides have very similar sequences and compositions, the significantly greater toxicity of LSP1 relative to LSP2 may be due to the relatively higher DBCO concentration in LSP1 (Figure S2). Therefore, we selected LSP2 and a concentration of 1 mg/mL for the following cell experiments. For simplicity, we referred to the LSP2 peptide as LSP in the remainder of this text. We also termed the coacervates formed by the LSP2 peptide Lysosome‐Sorting Peptide Coacervates (**LSP‐Coa)** (Table 1).

**TABLE 1:** Acronyms to full form table.

**Acronyms**	**Full form**
LSP	Lysosome‐Sorting peptide
**LSP‐Coa**	Lysosome‐Sorting peptide coacervates
BSA‐LSP	Bovine serum albumin LSP conjugates
GFP‐LSP	Green fluorescent protein LSP conjugates
Tras‐LSP	Trastuzumab LSP conjugates
Tras‐LSP/**LSP‐Coa**	**LSP‐Coa** containing Tras‐LSP
Ctx‐LSP	Cetuximab LSP conjugates
Ctx‐LSP/**LSP‐Coa**	**LSP‐Coa** containing Ctx‐LSP
**CoaLPD**	Coacervate‐mediated lysosome‐targeting protein degradation

Next, we used a fluorescent probe with a reported lifetime to assess the fluorescence lifetime of coacervates [43], showing quantitative evidence that the LSP1 and LSP2 coacervates had similar fluidity (Figure S3). Characterization of **LSP‐Coa** showed typical features of micron‐sized particles in PBS buffer and 10% FBS cell culture medium. Higher concentrations and neutral pHs promoted phase separation and coacervate formation. Furthermore, we quantified the dielectric constant and microviscosity of **LSP‐Coa** using the environmentally responsive fluorescent probes, 7‐sulfonamide benzoxadiazole fluorophore (SBD) and boron dipyrromethene fluorophore (BODIPY), respectively [44] (Figure S4). Based on the fluorescence lifetime of SBD, we calculated the dielectric constant ε of **LSP‐Coa** to be 57.5. Similarly, the fluorescence lifetime of BODIPY yielded a microviscosity η of 0.97 Pa·s. Regarding the stability of the coacervates, confocal imaging and turbidity analyses showed that the coacervates maintained droplet morphology for up to 24 h in both PBS and 10% FBS at 37°C (Figures S5 and S6). Importantly, no aggregation was observed at 4°C for at least five days.

### 2.2 Cellular Uptake and Lysosome Targeting of Peptide Coacervates

**FIGURE 2:**
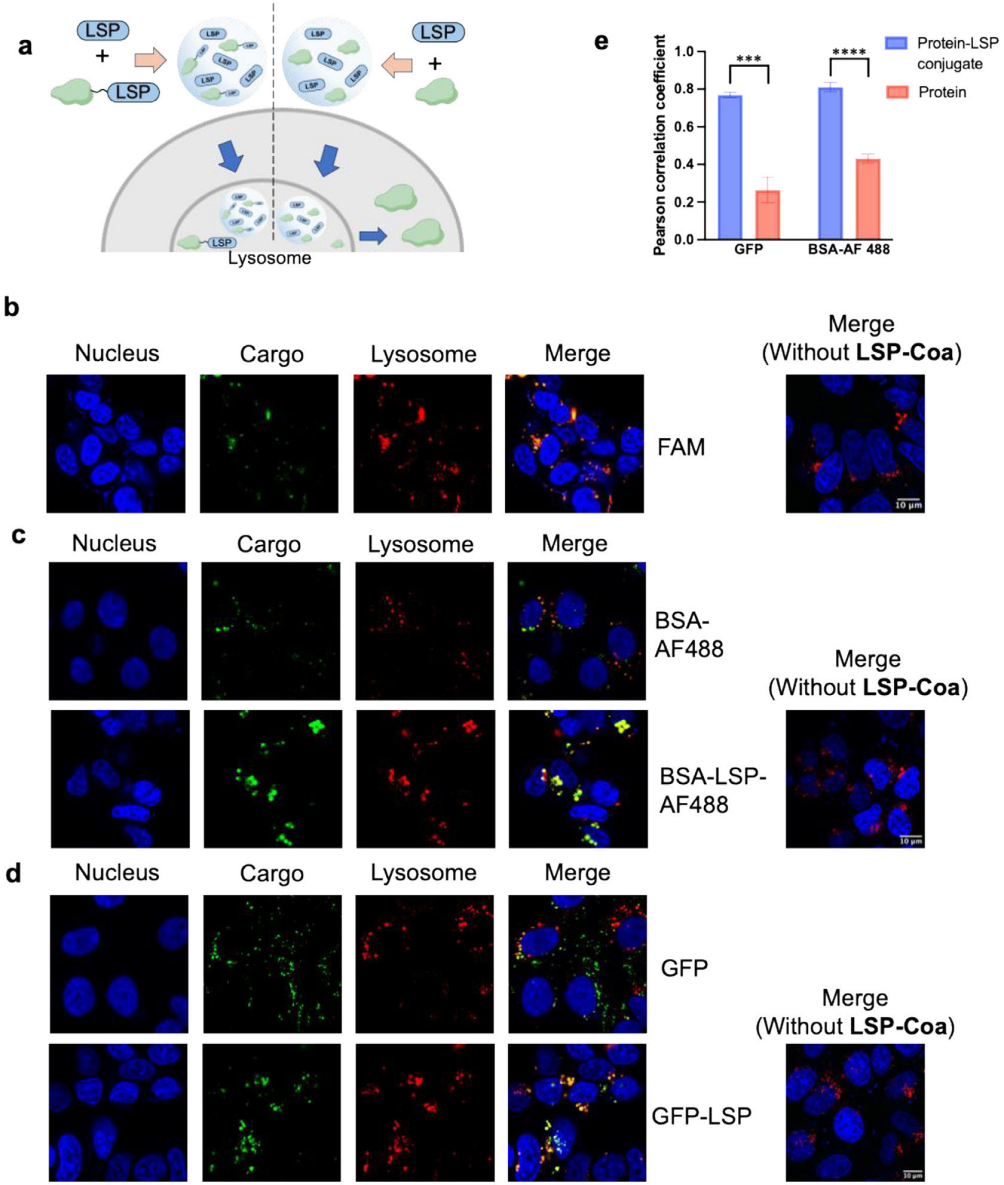
**LSP‐Coa** spontaneously enter cells and deliver proteins to the lysosome. (a) Scheme showing **LSP‐Coa**‐mediated lysosomal delivery of native and LSP‐conjugated proteins. (b) Confocal fluorescent images showing overlap of the FAM and LysoTracker signals in SK‐BR‐3 cells, indicating the colocalization of **LSP‐Coa** and lysosomes. Lysosome‐specific delivery of BSA and BSA‐LSP (c) and GFP and GFP‐LSP (d) by **LSP‐Coa**. Blue: Hoechst; Green: different cargoes; Red: LysoTracker Deep Red. (e) Pearson correlation coefficient values between the LysoTracker and protein signals within the cells treated by protein‐loaded coacervates or protein‐LSP‐loaded coacervates. Data are presented as the mean ± s.d. of n = 3 independent experiments. ^***^ *p* < 0.001, ^****^ *p* < 0.0001 according to two‐sided Student's t‐test.

We then examined protein encapsulation within **LSP‐Coa**. We observed that a range of fluorescent proteins could be encapsulated within **LSP‐Coa**, with calculated encapsulation efficiencies exceeding 60% (0.01 mg/mL protein to 1 mg/mL LSP peptide) (Figure S7). We then tested whether **LSP‐Coa** could deliver protein cargo into the cell and, specifically, to the lysosome. In particular, we compared the delivery of two forms of proteins: the native, unmodified form and the LSP‐conjugated form (Figure 2a). We envisioned that **LSP‐Coa** could deliver both native, unmodified proteins and LSP‐conjugated proteins to lysosomes, with the LSP‐conjugated proteins exhibiting greater lysosomal targeting efficiency. Next, we showed that LSP2 coacervates labeled with the fluorescent dye 6‐carboxyfluorescein (FAM) could spontaneously enter SK‐BR‐3 cells and colocalize with a lysosome‐specific dye, LysoTracker (Figure 2b). According to these data, we verified that LSP2 coacervates had a lysosome‐targeting feature inside cells. Next, we investigated whether **LSP‐Coa** could deliver protein cargo to the lysosome. We selected Alexa Fluor 488 (AF488)‐labeled bovine serum albumin (BSA) and green fluorescent protein (GFP) as the cargo. After incubating the protein‐encapsulated **LSP‐Coa** with SK‐BR‐3 cells for 12 h at 37°C, confocal microscopy revealed that the coacervates could bring both proteins into cells, and some proteins could colocalize with lysosomes, as indicated by signal overlap with LysoTracker (Figure 2c,d). To further enhance lysosomal targeting, we conjugated the LSP peptide to the cargo protein, yielding LSP‐conjugated BSA (BSA‐LSP‐AF488) and GFP (GFP‐LSP). Incubating LSP‐conjugated proteins and **LSP‐Coa** with cancer cells resulted in significant overlap of the cargo signal with the LysoTracker signal (Figure 2c–e), indicating that covalent conjugation of the cargo protein with LSP enhanced lysosome targeting. Interestingly, we found that the coacervates colocalized with Rab7a, a late endosomal marker, but not with Rab5a, an early endosomal marker (Figure S8), suggesting that the coacervates did not follow the canonical endocytic pathway to reach the lysosome. We then used the **LSP‐Coa** system to deliver protein cargoes to different cell lines. The results showed that the **LSP‐Coa** could deliver protein cargoes to both cancer and non‐cancer cell lines (Figure S9).

Next, we used endocytosis inhibitors targeting various endocytosis pathways, including chloroquine (an inhibitor of clathrin‐mediated endocytosis), dynasore (an inhibitor of dynamin‐mediated endocytosis), amiloride (an inhibitor of pinocytosis), 2‐deoxy‐D‐glucose (an inhibitor of energy‐dependent endocytosis), and cytochalasin B (an inhibitor of pinocytosis and phagocytosis), to suppress **LSP‐Coa** uptake (Figure S10). The cytochalasin B‐treated group gave the most significant inhibition of coacervate uptake, whereas chloroquine, dynasore, and amiloride treatments also inhibited coacervate uptake. Taken together, we revealed that coacervates may enter cells via multiple pathways, with macropinocytosis likely playing the most important role [45, 46].

### 2.3 LSP‐Coa‐mediated Degradation of Epidermal Growth Factor Receptors (EGFRs)

**FIGURE 3:**
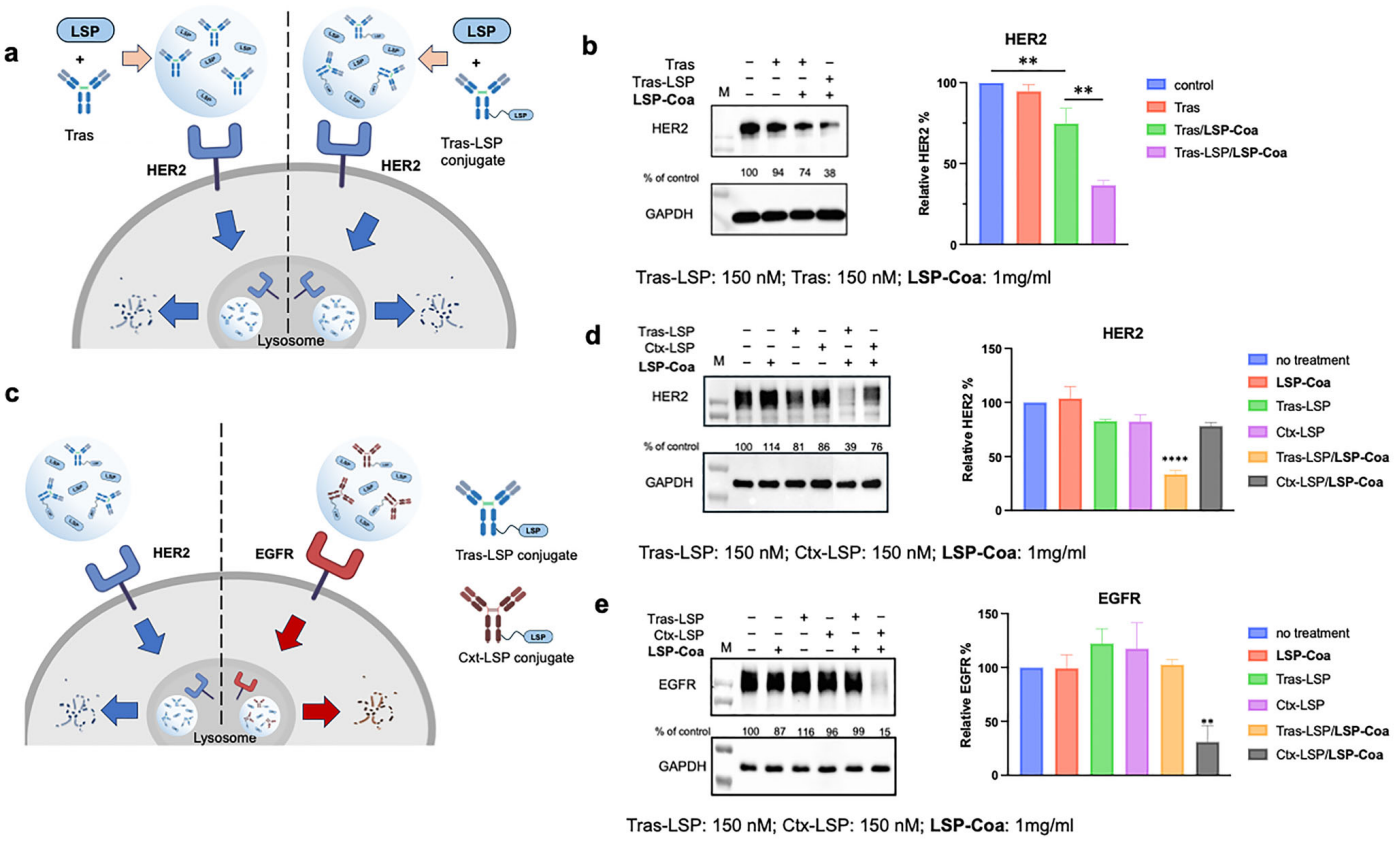
**LSP‐Coa**‐mediated cell surface antigens degradation. (a) Schematic illustration showing HER2 degradation using **LSP‐Coa** and Tras or Tras‐LSP. (b) Western blotting data revealing that the **LSP‐Coa** encapsulating Tras and Tras‐LSP could both lead to HER2 degradation, with the Tras‐LSP/**LSP‐Coa** treatment giving significantly higher HER2 knockdown. (c) Schematic illustration showing the respective degradation of HER2 or EGFR using Tras/**LSP‐Coa**, Tras‐LSP/**LSP‐Coa**, Ctx/**LSP‐Coa**, and Ctx‐LSP/**LSP‐Coa**. (d) Western blotting data revealing the Tras‐LSP/**LSP‐Coa** treatment leads to HER2 degradation, whereas the Ctx‐LSP/**LSP‐Coa** treatment leads to EGFR degradation. Protein levels were normalized using GAPDH as the control: relative HER2% = (HER2 signal_expt_ / GAPDH signal_expt_)/ (HER2 signal_no treatment_ / GAPDH signal_no treatment_)  × 100%; relative EGFR % = (EGFR signal_expt_ / GAPDH signal_expt_)/ (EGFR signal_no treatment_ / GAPDH signal_no treatment_) × 100%. Data are presented as the mean ± s.d. of n = 3 independent experiments. ^**^ *p* < 0.01, ^****^ *p* < 0.0001 according to two‐sided Student's t‐test, compared to the no‐treatment group.

Next, we employed the **LSP‐Coa** system to degrade the human epidermal growth factor receptor 2 (HER2) (Figure 3a). First, anti‐HER2 antibody trastuzumab (Tras) was modified with azide using an azide NHS ester and then conjugated with the LSP through the azide‐DBCO click reaction, resulting in Tras‐LSP conjugates (Figure S11). Then, Tras or Tras‐LSP were mixed with LSP to form Tras‐loaded coacervates (Tras/**LSP‐Coa**) or Tras‐LSP‐loaded coacervates (Tras‐LSP/**LSP‐Coa**, antibody: 150 nM; **LSP‐Coa**: 1 mg/mL). These coacervates were incubated with SK‐BR‐3 cells for 16 h at 37°C. The cells were subsequently lysed, and the lysates were analyzed by Western blotting to determine HER2 levels. The results indicated that both two coacervates could lead to HER2 degradation, with the Tras‐LSP/**LSP‐Coa** treatment giving a significantly higher degradation efficiency (Figure 3b), indicating **LSP‐Coa** could transfer the HER2–Tras‐LSP complexes into cells and target the lysosome for degradation. Covalent conjugation of the Tras with the LSP peptide significantly enhanced the degradation efficiency. The degradation efficiency of HER2 was related to the Tras‐LSP concentration and incubation time (Figure S12). The immunofluorescent data also showed a significant decrease in the HER2 signal following Tras‐LSP/**LSP‐Coa** treatment (Figure S13). Therefore, here we present a previously unreported approach of protein degradation, termed coacervate‐mediated lysosome‐targeting protein degradation, or **CoaLPD**.

Next, we tested the target specificity of **CoaLPD**. The anti‐EGFR (epidermal growth factor receptor) antibody cetuximab (Ctx) was conjugated with LSP using the same method as the Tras‐LSP conjugate to form the Ctx‐LSP conjugate. These two conjugates were mixed with the LSP peptide to form Tras‐LSP/**LSP‐Coa** and Ctx‐LSP/**LSP‐Coa** (antibody‐LSP conjugates: 150 nm; **LSP‐Coa**: 1 mg/mL). The coacervates were incubated with SK‐BR‐3 cells for 16 h at 37°C, and Tras‐LSP and Ctx‐LSP without coacervates were used as controls (Figure 3c). The cells were subsequently lysed, and the lysates were analyzed by Western blotting to determine HER2 and EGFR levels. The results indicated that treatment with Tras‐LSP/**LSP‐Coa** reduced HER2 expression by approximately 60% compared to the negative control, whereas the Ctx‐LSP/**LSP‐Coa** treatment did not cause HER2 decrease (Figure 3d). Similarly, Ctx‐LSP/**LSP‐Coa** treatment reduced EGFR levels by 80% but did not knock down HER2 (Figure 3e). These data manifest the selectivity of targeted protein degradation.

### 2.4 Simultaneous Knockdown of Two Cancer‐associated Proteins

**FIGURE 4:**
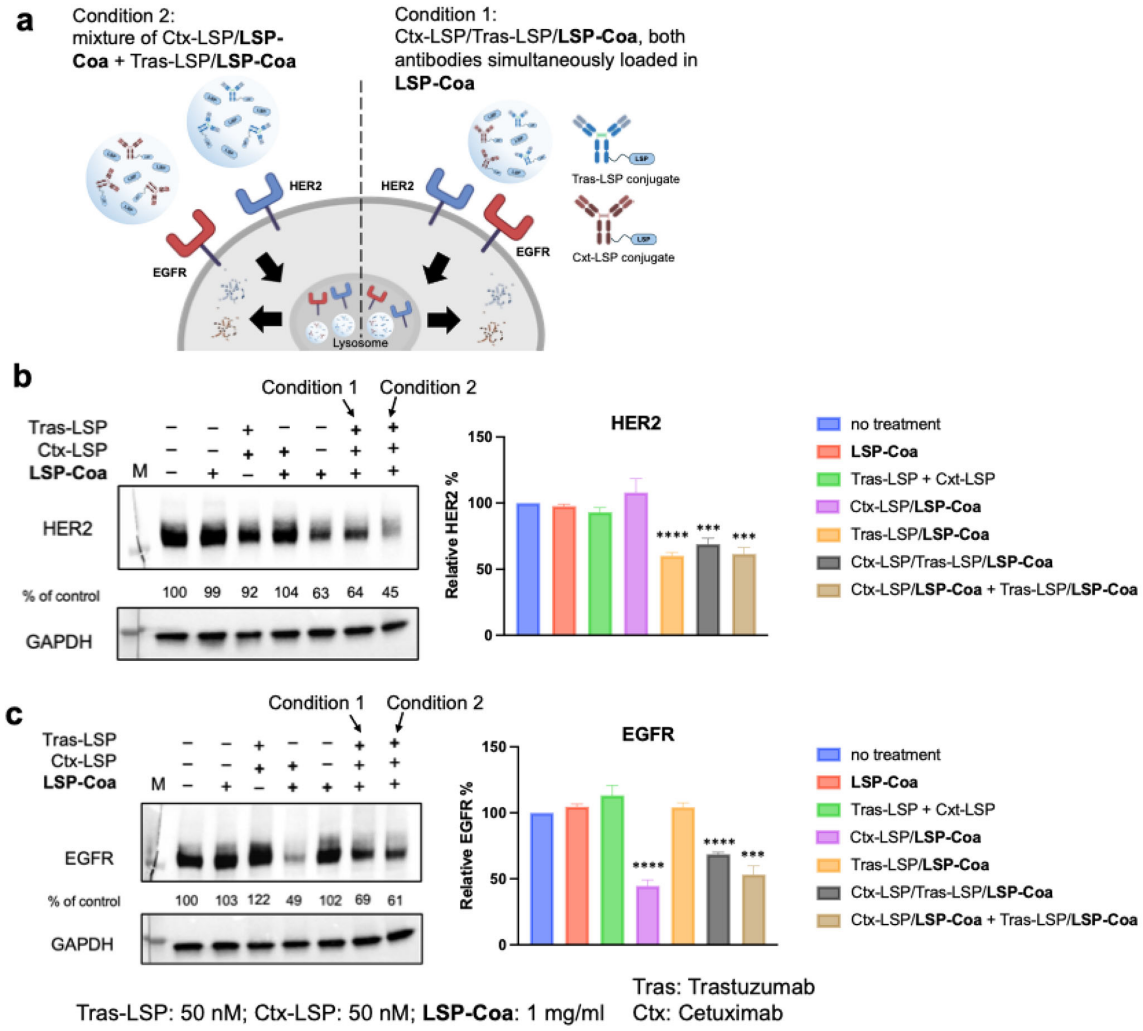
Simultaneous knockdown of two receptors, HER2 and EGFR. (a) Scheme of the **LSP‐Coa**‐mediated simultaneous degradation of both EGFR and HER2. (b) Western blot showing Tras‐LSP/**LSP‐Coa** + Ctx‐LSP/**LSP‐Coa** and Tras‐LSP/Ctx‐LSP/**LSP‐Coa** could lead to HER2 degradation with the quantitative analysis. (c) Western blot showing Tras‐LSP/**LSP‐Coa** + Ctx‐LSP/**LSP‐Coa** and Tras‐LSP/Ctx‐LSP/**LSP‐Coa** could lead to EGFR degradation with the quantitative analysis. Protein levels were normalized using GAPDH as the control: Relative HER2% = (HER2 signal_expt_ / GAPDH signal_expt_)/ (HER2 signal_no treatment_ / GAPDH signal_no treatment_)  × 100%; relative EGFR % = (EGFR signal_expt_ / GAPDH signal_expt_)/ (EGFR signal_no treatment_ / GAPDH signal_no treatment_) × 100%. Data are presented as the mean ± S.D. of n = 3 independent experiments. ^***^ *p* < 0.001, ^****^ *p* < 0.0001 according to two‐sided Student's t‐test, compared to the no‐treatment group.

Next, we show that the **CoaLPD** strategy is amenable to degrading HER2 and EGFR simultaneously. Two variations were developed. In the first method, we combined Tras‐LSP, Ctx‐LSP, and LSP to create coacervates that encapsulate both antibodies, which we refer to as Tras‐LSP/Ctx‐LSP/**LSP‐Coa** (condition 1) (Tras‐LSP: 50 nM; Ctx‐LSP: 50 nm; **LSP‐Coa**: 1 mg/mL) (Figure 4a). Alternatively, we pre‐formed Tras‐LSP/**LSP‐Coa** (Tras‐LSP: 50 nM; **LSP‐Coa**: 1 mg/mL) and Ctx‐LSP/**LSP‐Coa** (Ctx‐LSP: 50 nM; **LSP‐Coa**: 1 mg/mL) and then added these two coacervates to the cells (Tras‐LSP/**LSP‐Coa**+ Ctx‐LSP/**LSP‐Coa**) (condition 2) (Figure 4b). After 16‐h incubation, cell lysates were collected and analyzed by Western blotting to assess HER2 and EGFR levels. Both strategies successfully degraded HER2 and EGFR (Figure 4b,c). These results demonstrate that lysosomal‐targeting peptide coacervates, combining LSP‐conjugated antibodies, can effectively degrade cell surface cancer‐associated proteins through various combinations. Next, we compared these two conditions. Using BSA‐LSP proteins covalently labeled with two fluorescent dyes, AF488 and Cy5, we observed that in BSA‐LSP‐AF488/BSA‐LSP‐Cy5/**LSP‐Coa** (condition 1), a significant overlap of both fluorescent signals was found (Pearson correlation coefficient measured to be 0.708). Or, within 10 min of mixing BSA‐LSP‐AF488/**LSP‐Coa** with BSA‐LSP‐Cy5/**LSP‐Coa** (condition 2), the two fluorescent signals started to overlap, indicating a fast droplet fusion in the solution (Figure S14a). The Tras‐LSP‐AF488/Ctx‐LSP‐Cy5/**LSP‐Coa** (condition 1) solution showed a similar result (Figure S14b). In cells, however, the droplet fusion is much slower. While incubation of BSA‐LSP‐AF488/BSA‐LSP‐Cy5/**LSP‐Coa** with SK‐BR‐3 cells showed a significant overlap of both fluorescent signals (Pearson correlation coefficient of about 0.696), the mixture of BSA‐LSP‐AF488/**LSP‐Coa** and BSA‐LSP‐Cy5/**LSP‐Coa** (condition 2) in cells showed a rather slow droplet fusion after 12 h of incubation (Pearson correlation coefficient of about 0.246) (Figure S14c). These data show that the crowded cytosolic environment slows droplet fusion, but both conditions can lead to marked degradation of targeted proteins.

Degrading two CAPs simultaneously may reduce the risk of drug resistance. Therefore, we did the cell proliferation experiments. We treated the cells with 100 nm Ctx‐LSP/**LSP‐Coa**, 100 nm Tras‐LSP/**LSP‐Coa**, 50 nm Tras‐LSP/**LSP‐Coa** + 50 nm Ctx‐LSP/**LSP‐Coa**, and 50 nm Ctx + 50 nm Tras as the control group on day 0 and day 2. The number of cells was tested daily using CCK8, and cell numbers were normalized using the no‐treatment group as the control. From the results, we could find that treating cancer cells with Tras‐LSP/**LSP‐Coa** or Ctx‐LSP/**LSP‐Coa** could effectively inhibit their proliferation, and the inhibitory effect of treating cancer cells with Tras‐LSP/**LSP‐Coa** and Ctx‐LSP/**LSP‐Coa** at the same time was significantly better than using a single antibody (Figure S15).

### 2.5 Lysosomal Degradation Pathway

**FIGURE 5:**
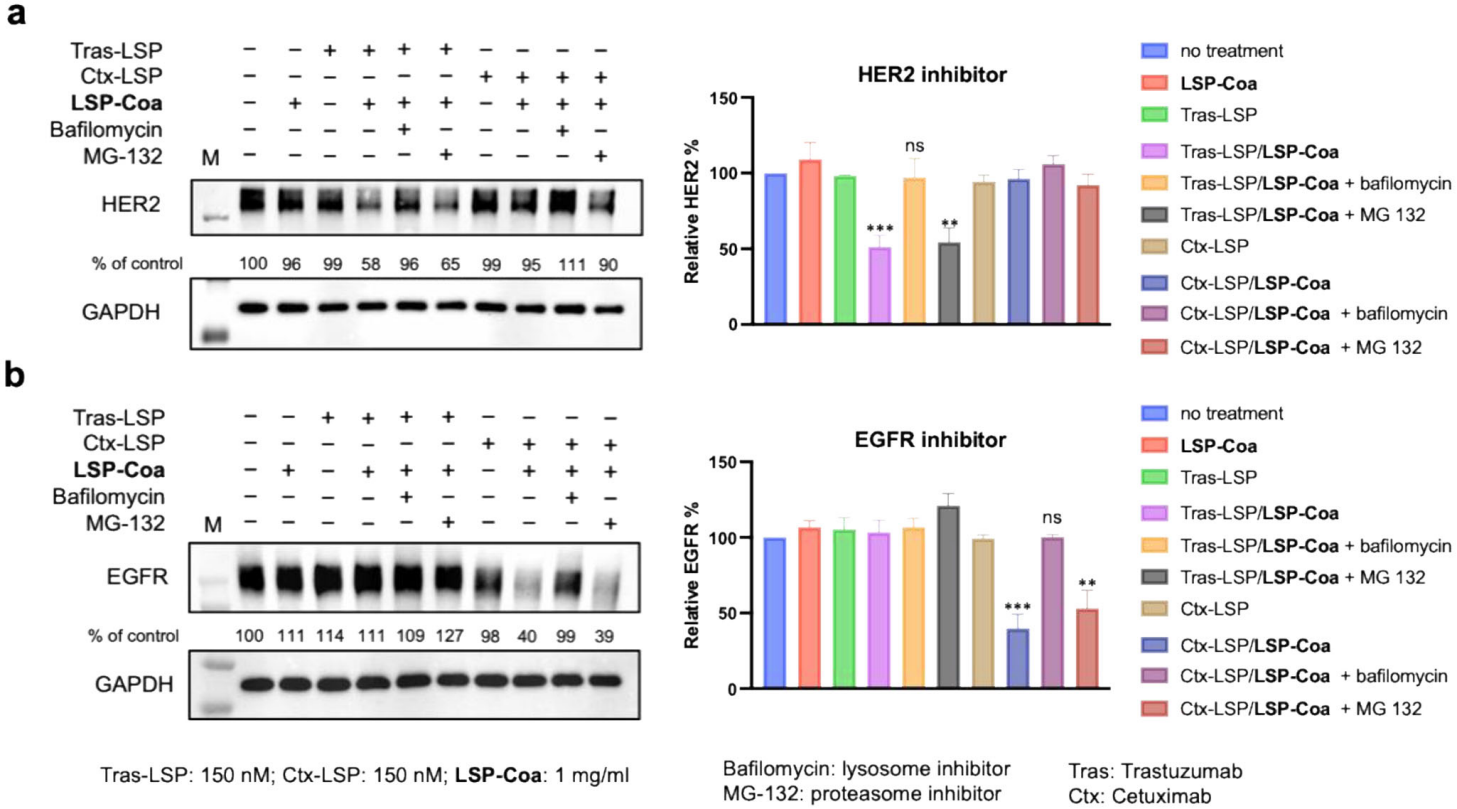
Lysosome inhibitor bafilomycin blocks the antibody‐mediated **CoaLPD**. (a) HER2 Western blot (left) of SK‐BR‐3 cells treated with Tras‐LSP/**LSP‐Coa** and Ctx‐LSP/**LSP‐Coa** for 16 h along with 100 nm MG132 or 100 nm bafilomycin and quantitative analysis of the Western blot (right) (b) EGFR Western blot (left) of SK‐BR‐3 cells treated with Tras‐LSP/**LSP‐Coa** and Ctx‐LSP/**LSP‐Coa** for 16 h along with 100 nm MG132 or 100 nm bafilomycin and quantitative analysis of the Western blot (right). Protein levels were normalized using GAPDH as the control: Relative HER2% = (HER2 signal_expt_ / GAPDH signal_expt_)/ (HER2 signal_no treatment_ / GAPDH signal_no treatment_)  × 100%; relative EGFR % = (EGFR signal_expt_ / GAPDH signal_expt_)/ (EGFR signal_no treatment_ / GAPDH signal_no treatment_) × 100%. Data are presented as the mean ± s.d. of n = 3 independent experiments. ^**^ *p* < 0.01, ^***^ *p* < 0.001 according to two‐sided Student's t‐test, compared to the no‐treatment group.

Next, we showed that **CoaLPD** mainly adopted the lysosome pathway instead of the ubiquitin/proteasome pathway. We co‐incubated the coacervates and 100 nm proteasome inhibitor, MG132, or 100 nm lysosome inhibitor, bafilomycin, respectively, with cells. The degradation of HER2 and EGFR (Figure 5a,b) was significantly blocked by bafilomycin but not by MG132. These results were consistent with the confocal images (Figure S16), which showed colocalization of the fluorescence‐labeled antibodies and lysosomes. On the other hand, we used confocal microscopy to observe the colocalization of antibodies and lysosomes at different time points after the addition of Tras‐LSP/**LSP‐Coa**. The results showed that as the incubation time increased, the colocalization signal between antibodies and lysosomes also strengthened (Figure S17). These data suggest that in **CoaLPD**, the targeted proteins are most likely degraded through the lysosomal pathway.

### 2.6 LSP‐Coa Enhances the Efficiency of a PROTAC

**FIGURE 6:**
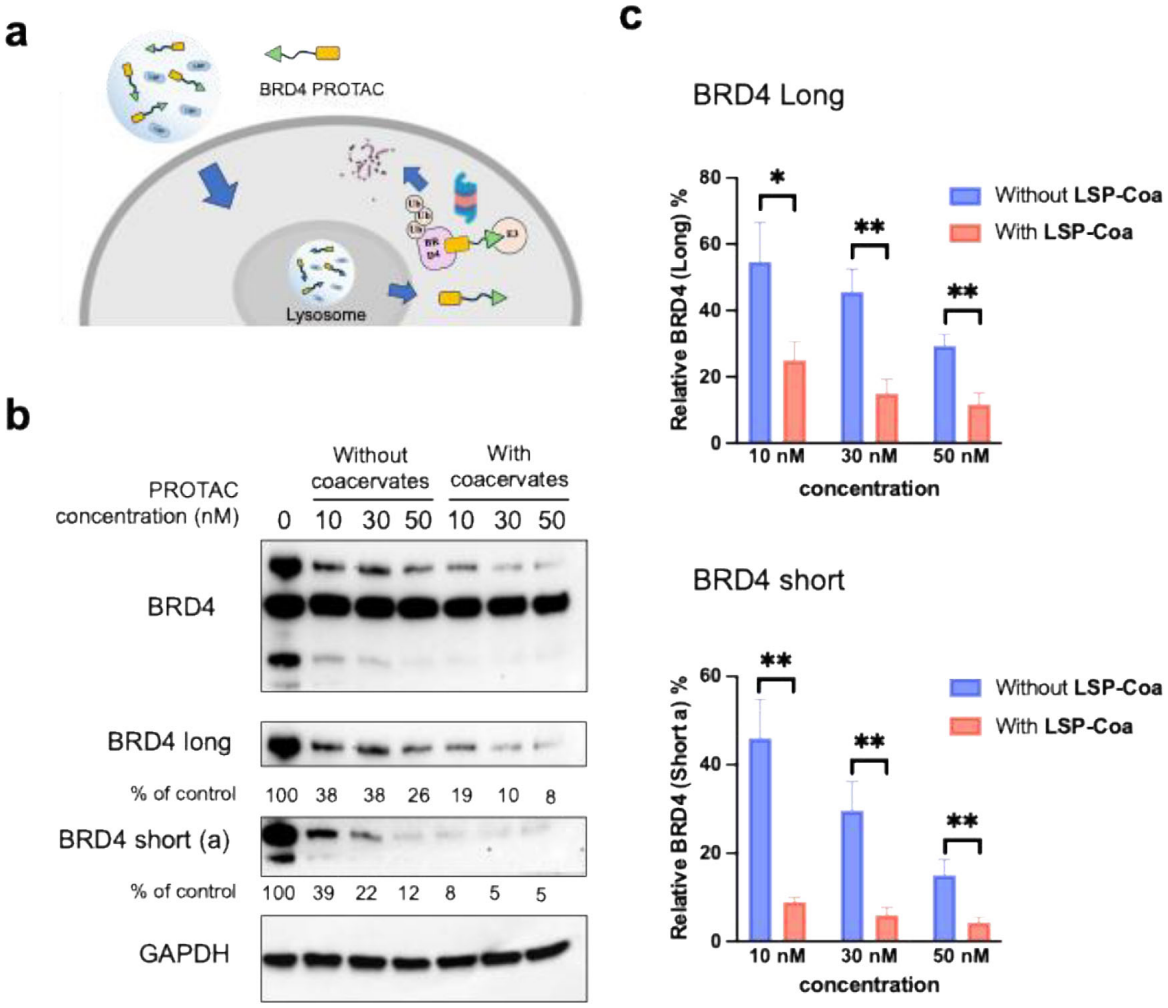
**LSP‐Coa** enhances PROTAC degradation efficiency. (a) Scheme of the **LSP‐Coa**‐facilitated BRD4 PROTAC degradation. (b) Western blotting data showing **LSP‐Coa** enhances the degradation of both long and short forms of BRD4. Quantitative analysis of the Western blot: (up) relative BRD4 long % = (BRD4 long signal_expt_ / GAPDH signal_expt_)/ (BRD4 long signal_no treatment_ / GAPDH signal_no treatment_) × 100%; (down) BRD4 short (a) % = (BRD4 short (a) signal_expt_ / GAPDH signal_expt_)/ (BRD4 short (a) signal_no treatment_ / GAPDH signal_no treatment_) × 100%. Data are presented as the mean ± S.D. of n = 3 independent experiments. ^*^ *p* < 0.05, ^**^ *p* < 0.01 according to two‐sided Student's t‐test.

Next, we examined whether **LSP‐Coa** can increase the efficiency of other degraders by enhancing their cellular uptake (Figure 6a) [47, 48]. Using the PROTAC BRD4 Degrader‐5 as a representative [49], we incubated the BRD4 PROTAC molecule (10, 30, or 50 nm) and SK‐BR‐3 cells with or without **LSP‐Coa** (1 mg/mL) for 12 h. We then lysed the cells to measure the BRD4 level by Western blotting. The BRD4 PROTAC decreased BRD4 levels and reduced the expression level of the long form at 30 nm without **LSP‐Coa**. However, co‐incubation with **LSP‐Coa** decreased the BRD4 long form to about 30% at 30 nm of PROTAC (Figure 6b). A similar trend has been found in the BRD4 short form: at 30 nm, 70% reduction without **LSP‐Coa** vs. 95% reduction with **LSP‐Coa** (Figure 6c).

In addition, we labeled PROTAC molecules with a fluorescent dye and tracked the location of PROTAC molecules after delivery into cells by **LSP‐Coa** using confocal microscopy. Confocal microscopy images showed that PROTAC molecules had high colocalization with lysosomes 2 h after adding **LSP‐Coa**, indicating that **LSP‐Coa** could deliver PROTAC molecules into lysosomes. After extending the incubation time to 6 h, PROTAC molecules showed more colocalization with the nucleus, suggesting that PROTAC molecules escaped from lysosomes and reached the nucleus to exert their effects (Figure S18). Next, we added into the system 100 nm MG132 or 100 nm bafilomycin to block the protease pathway or the lysosome pathway, respectively (Figure S19). We found that PROTAC‐mediated BRD4 degradation was significantly inhibited by MG132 but not by bafilomycin. These findings suggest that **LSP‐Coa** promotes PROTAC degradation by enhancing its cellular uptake without changing the ubiquitin/protease‐dependent degradation pathway. We speculate that the PROTAC molecules may have entered the lysosome and escaped into the cytosol (Figure 6a).

### 2.7 Anticancer Effect of CoaLPD In Vivo

**FIGURE 7:**
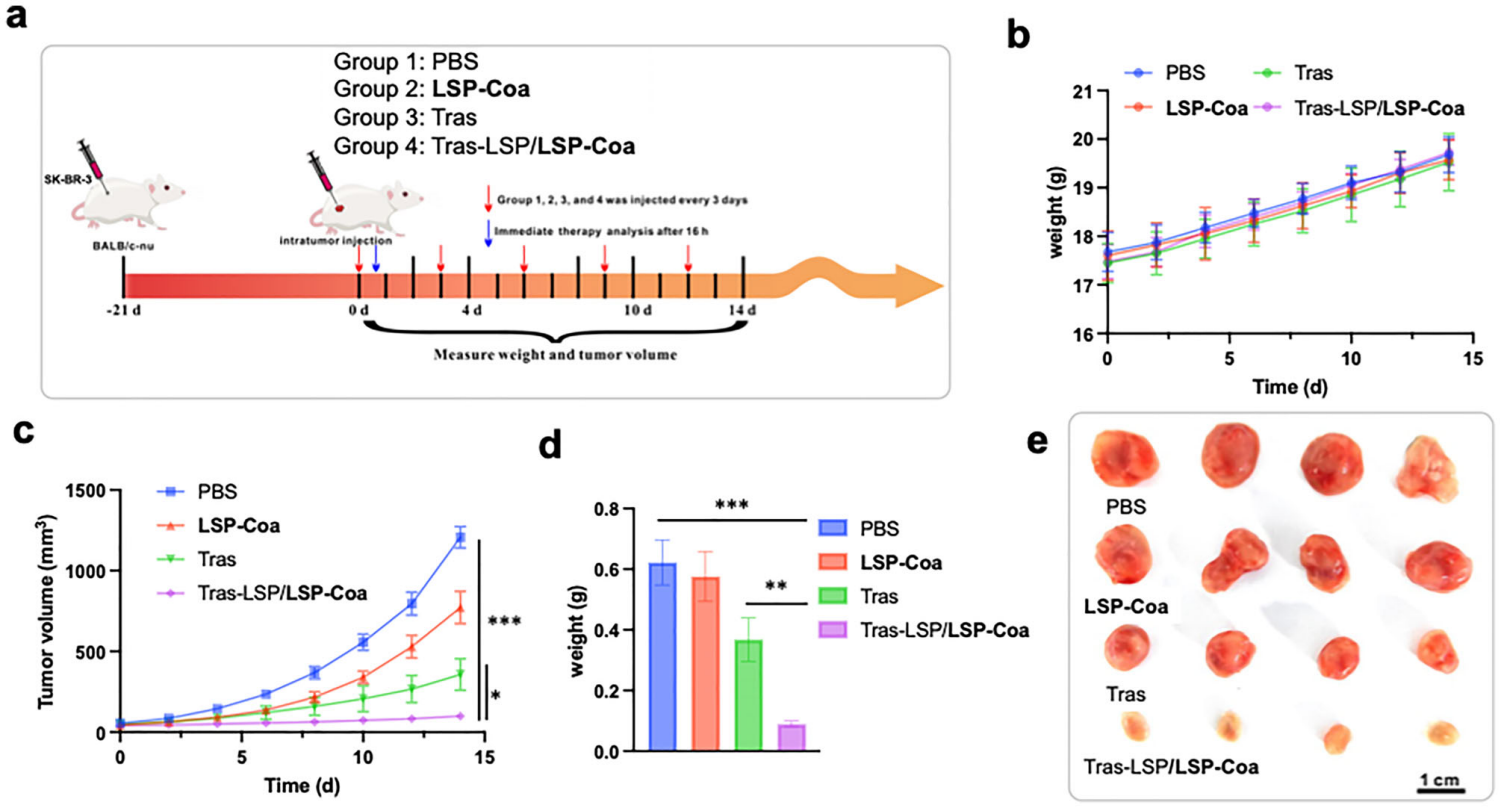
Anti‐tumor therapeutic effect of Tras‐LSP/**LSP‐Coa**‐mediated HER2 degradation in vivo. (a) Scheme showing the workflow of the anti‐tumor treatment experiments. Briefly, SK‐BR‐3 cells (1 × 10^6^) were injected subcutaneously into 5‐week‐old BALB/c female nude mice. Drugs were administered intratumorally from day 0 and injected every 3 days. (b) Body weights of different groups of mice were monitored during the treatment period. (c) Tumor volumes of different groups of mice were measured during the treatment periods. (d) Tumor weight comparison after the mice were sacrificed on Day 14. (e) Comparison of dissected tumors after the mice were sacrificed on Day 14. Data are presented as the mean ± S.D. of n = 3 independent experiments. ^*^ *P*<0.05, ^**^ *p* < 0.01, ^***^ *p* < 0.001 according to two‐sided Student's t‐test.

Lastly, we demonstrated the anti‐tumor potential of **CoaLPD** using Tras‐LSP/**LSP‐Coa** to kill HER2+ tumors in vivo. We established a mouse model of breast cancer by subcutaneously inoculating SK‐BR‐3 cells into 5‐week‐old BALB/c female nude mice. When the tumor size reached 40–50 mm^3^, we intratumorally injected 20 µL PBS, **LSP‐Coa** (5 mg/mL), Tras (4 µg), or Tras‐LSP/**LSP‐Coa** (5 mg/mL LSP containing 4 µg Tras‐LSP) into the mice every three days for 12 days (Figure 7a). These injections did not cause significant weight changes in any of the treatment groups (Figure 7b). Tras‐LSP/**LSP‐Coa** injection exhibited the most substantial tumor growth inhibition, evidenced by a significant decrease in both tumor size and weight compared to other groups (Figure 7c–e; Figure S20). Additionally, TUNEL and HE staining of tumor tissues revealed considerable cell necrosis in the Tras‐LSP/**LSP‐Coa** treatment group (Figure S21a), and the tumor tissue immunofluorescence analysis showed the lowest HER2 level in the Tras‐LSP/**LSP‐Coa** group (Figure S21b), both suggesting that the Tras‐LSP/**LSP‐Coa** treatment could inhibit the HER2+ tumor growth by degrading HER2 in vivo.

## 3 Experimental Section

### 3.1 Antibody Conjugation Reactions

Briefly, 150 µL of a 2.0 mg/mL stock of antibody (Trastuzumab or cetuximab) was added with 15 µL of 1.0 m NaHCO_3_ and 10 µL of a 10 mm stock of Azide PEG_3_ NHS ester (Leyan, HY‐126528) in DMSO. The mixture was incubated at room temperature for 1.5 h. Then, the reaction mixture was passed through a desalting column (Zeba Spin Desalting Columns, 7K MWCO, 0.5 mL), pre‐equilibrated with PBS buffer (pH 7.4, 137 mM NaCl, 2.7 mM KCl, 10 mM Na_2_HPO_4_, 1.8 mm KH_2_PO_4_). For the antibody‐LSP conjugation, the final concentration of the antibody was 0.75 µm, and the LSP peptide was 75 µm in PBS buffer for 1 h. After the reaction, the mixture was denatured in the presence of loading dye and heated at 95°C for 10 min before SDS‐PAGE analysis.

### 3.2 LSP‐Coa Formation

The peptide was first dissolved at 100 mg/mL in DMSO. We then diluted the stock solution in PBS or cell culture medium at 1:100. For the fusion and FRAP experiments, the peptide was added to PBS buffer containing 0.1 mg/mL of Nile Red. For the degradation experiment, the azide‐modified antibody (Tras‐N_3_ or Ctx‐N_3_) was diluted to 100 µL in cell culture medium without FBS, and then 1 µL of the peptide stock solution in DMSO was added to the mixture. After 30 min incubation, 4 µL 100 mg/mL LSP peptide stock was added to the solution, and 400 µL cell culture medium with 10% FBS was added to form the Tras‐LSP/**LSP‐Coa** or Ctx‐LSP/**LSP‐Coa** complexes.

### 3.3 Calculations of Physical Parameters

For the measurement of the dielectric constant, the fluorescence lifetime (τ) of LSP2 coacervates (**LSP‐Coa**) labeled with SBD was measured by fluorescence lifetime imaging microscopy (FLIM). Based on the following equation, τ  =   − 10.78*log*_10_ε + 20.25, the dielectric constant ε was calculated to be 57.5 [44]. For microviscosity measurements, the fluorescence lifetime of **LSP‐Coa** labeled with BODIPY was determined using FLIM. Based on the following equation, τ  =  1.35*log*_10_η + 0.27, microviscosity η was calculated to be 0.97 Pa·s [44].

For the calculation of the diffusion coefficient, the recovery data of FRAP were normalized to the unbleached reference, and then the curve was fitted using a non‐linear exponential model: *y*  =  *y*0 + (*Plateau* − *y*0)*(1 − *e* ^−K*x^), in which y0 is the y value (normalized fluorescence intensity of the bleach area) when y (time) is zero; plateau is the y value at infinite times, K is the rate constant. For LSP1 coacervates, the fitting curve is *y*  =  14.1 + 90.5*(1 − *e* ^−0.081*x^). The t_1/2_ is 8.58 s. The diffusion coefficient was calculated using the Soumpasis equation: $D=0.224\frac{r^{2}}{t_{1/2}}$, in which r is the radius of the bleach area. The diffusion coefficient *D* of LSP1 coacervates is calculated to be 2.84 × 10^−2^ µm^2^/s. For LSP2 coacervates (**LSP‐Coa**), the fitting curve is *y*  =  19.25 + 87.65*(1 − *e* ^−0.031*x^). The time to half‐maximum (t_1/2_) value is 22.25 s. The diffusion coefficient D is about 4.33 × 10^−3^ µm^2^/s [42].

### 3.4 Cell Membrane Protein Degradation

5×10^5^ SK‐BR‐3 cells were seeded in a 12‐well plate (Wuxi NEST Biotechnology Co., Ltd) and incubated for 24 h in a humidified incubator (at 37°C, 5% CO_2_). Then, 500 µL Tras‐LSP/**LSP‐Coa** or Ctx‐LSP/**LSP‐Coa** complexes were added to the cells, and were incubated for 12 h. After washing the cells with PBS three times, 50 µL of radio‐immunoprecipitation assay (RIPA) lysis buffer was added to each well for cell lysis. The supernatants were collected for Western blot analysis.

### 3.5 Antitumor Therapy In Vivo

Approximately 1 × 10^6^ SK‐BR‐3 cells were inoculated subcutaneously into 5‐week‐old BALB/c female mice. After the tumors reached a defined volume, the mice were randomly assigned to 4 groups, with 5 mice per group. Then, 25 µL of different reagents were injected intratumorally. (I)PBS, (II) **LSP‐Coa**, (III) Tras, (IV) Tras‐LSP/**LSP‐Coa** (LSP: 10 mg/mL, Tras: 4 µg). We performed injections once every 3 days for 5 consecutive days, recorded weight and tumor volume changes using digital vernier calipers and scales every 2 days, and observed and photographed the dissected mouse tumors after 14 days. Additionally, one mouse tumor was collected from each group after 16 h for DAPI, TUNEL immunofluorescence, and HE staining; the slices were then examined under the microscope. All animal experiments were approved by the Animal Experimentation Ethics Committee of Huazhong University of Science and Technology (IACUC No. 4207).

### 3.6 Statistical Analysis

Statistical analyses were performed using GraphPad Prism 10. Unless otherwise stated, data are presented as mean ± standard deviation (S.D.) from n = 3 independent experiments. Western blot band intensities were quantified using ImageJ/Fiji, and target protein levels were normalized to GAPDH. Normalized values were then expressed as percentages relative to the no‐treatment control, as described in the figure captions. No data transformation was applied, and no outliers were excluded. Comparisons were conducted using a two‐sided unpaired Student's t‐test. A *p* value < 0.05 was considered statistically significant. Significance is denoted as: ^*^ *p* < 0.05, ^**^ *p* < 0.01, ^***^ *p* < 0.001, ^****^ *p* < 0.0001.

## 4 Conclusion

**SCHEME 1:**
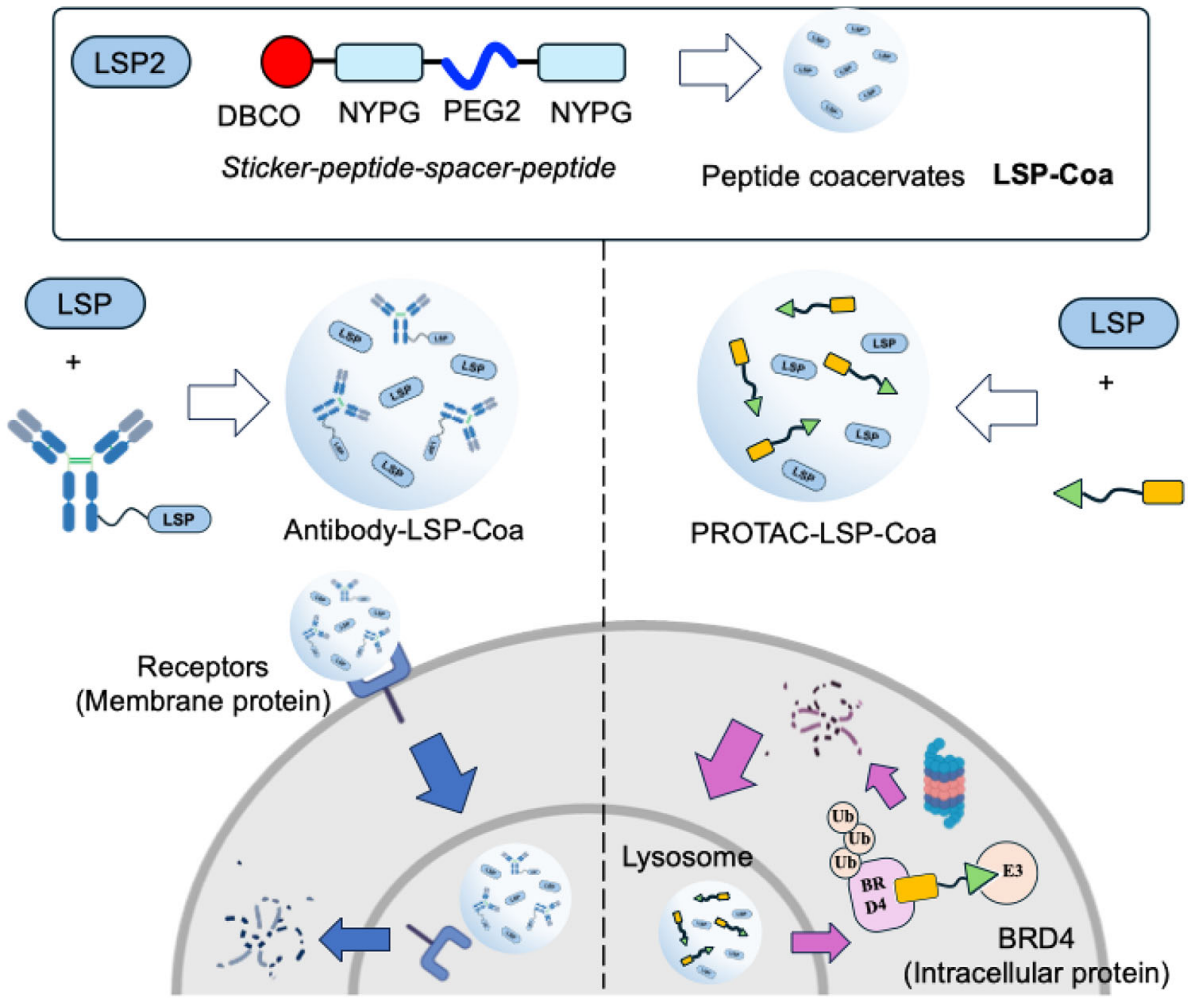
Schematic illustration showing the design of lysosome‐specific peptide coacervates for coacervate‐mediated lysosome‐targeting protein degradation.

As a promising anticancer therapy, targeted protein degradation relies on specific recognition of CAPs before transporting them to the cellular recycling machinery. While PROTAC technology utilizes small molecules to bind to the target protein, LYTAC employs antibodies to selectively identify the target protein. However, the protein complex formed by the antibody and the cell surface protein must be transported across the plasma membrane into cells and further translocated into the lysosome. Here, we report the development of peptide coacervates **LSP‐Coa** to achieve this. **LSP‐Coa**, in conjunction with LSP‐conjugated antibodies, achieved efficient and specific degradation of cancer‐associated cell surface proteins, enabling a receptor‐free LYTAC‐like protein degradation strategy that we termed **CoaLPD** (Scheme 1). After proving its selectivity, efficiency, and versatility in cells, we showed that the **CoaLPD** strategy incurred a pronounced anti‐tumor effect in vivo in an animal model of breast cancer. Furthermore, besides increasing antibody‐dependent protein degradation, **LSP‐Coa** also improved the degradation efficiency of a PROTAC molecule. Taken together, here we report a strategy for converting a lysosome‐specific tetrapeptide to form lysosome‐sorting peptide coacervates, and present coacervate‐induced internalization with the lysosome targeting property as a new strategy for targeted protein degradation as a potential anticancer treatment.

## Conflicts of Interest

The authors declare no conflicts of interest.

## Supporting information




**Supporting File**: advs74526‐sup‐0001‐SuppMat.pdf.

## Data Availability

The data that support the findings of this study are available from the corresponding author upon reasonable request.;
