# Supplementary material for: Coacervate‐Mediated Lysosome‐Targeting Antibody Delivery for Protein Degradation
Source: Adv Sci (Weinh). 2026 Feb 25;13(25):e20441. doi: 10.1002/advs.202520441 (PMC13137841; doi:10.1002/advs.202520441)
Supplement: Supplementary file 1 — Supporting File: advs74526‐sup‐0001‐SuppMat.pdf. [file ADVS-13-e20441-s001.pdf]

Supporting Information

Coacervate-Mediated Lysosome-Targeting Antibody Delivery for Protein Degradation

Dingdong Yuan,[a] Yishu Bao,[a] Zhiyi Xu,[a] Zhong Zheng,[a] Yongxu Han,[a] Kai Cheng,*,[a] Yuan-Di Zhao,*,[b] Jiang Xia*,[a]

[a] Department of Chemistry, The Chinese University of Hong Kong, Shatin, Hong Kong SAR 99999, China.

[b] Britton Chance Center for Biomedical Photonics at Wuhan National Laboratory for Optoelectronics-Hubei Bioinformatics & Molecular Imaging Key Laboratory, Department of Biomedical Engineering, College of Life Science and Technology, Huazhong University of Science and Technology, Wuhan 430074, Hubei, China.

*Address correspondence to Kai Cheng (kaicheng@cuhk.edu.hk), Yuan-Di Zhao (zydi@mail.hust.edu.cn, ORCID 0000-0002-4286-4275), and Jiang Xia (jiangxia@cuhk.edu.hk, ORCID 0000-0001-8112-7625)

Phone: (852) 3943 6165

Fax: (852) 2603 5057

Contents

**Items**	**Page No.**
**Detailed experimental procedures**	**S5**
**Table S1**. Screening of peptide derivatives for coacervate states.	**S10**
**Figure S1**. Cytotoxicity of LSP1 and LSP2.	**S11**
**Figure S2.** Cytotoxicity of DBCO-containing compounds.	**S12**
**Figure S3**. Qualitative evaluation of the fluidity of peptide coacervates.	**S13**
**Figure S4.** Physical properties of **LSP-Coa**.	**S14**
**Figure S5.** Confocal fluorescent images of **LSP-Coa** maintained in PBS or 10% FBS for 96 hours.	**S15**
**Figure S6.** Stability of **LSP-Coa** under different conditions.	**S16**
**Figure S7.** Encapsulation of different fluorescent proteins or fluorescent dyes in the **LSP-Coa**.	**S17**
**Figure S8.** Confocal fluorescent images showing the co-localization of **LSP-Coa**/protein complex with late endosomes.	**S18**
**Figure S9.** Protein delivery to different cell lines using **LSP-Coa**.	**S19**
**Figure S10**. Flow cytometry data showing inhibition of **LSP-Coa** internalization by endocytosis inhibitors.	**S20**
**Figure S11**. LSP-antibody conjugation.	**S21**
**Figure S12.** Dose- and time-dependent **LSP-Coa**-mediated degradation of cell-surface antigens.	**S22**

**Figure S13.** Immunofluorescent images showing down-regulation of HER2 on SK-BR-3 cells after different degradation through the confocal microscope.	**S23**
**Figure S14.** Comparison of the two conditions *in vitro* and in cells.	**S24**
**Figure S15.** Inhibition of cell proliferation using antibody-LSP/**LSP-Coa**.	**S26**
**Figure S16.** Confocal fluorescent images for co-localization of the antibody and different organelles.	**S27**
**Figure S17.** Confocal fluorescent images for Tras-LSP-AF488 delivery to SK-BR-3 cells through **LSP-Coa** with different incubation times.	**S28**
**Figure S18.** Confocal fluorescent images for PROTAC location in cells.	**S29**
**Figure S19.** Mechanism of BRD4 degradation with or without **LSP-Coa**.	**S30**
**Figure S20.** Tumor-to-body weight ratio for *in vivo* experiments.	**S31**
**Figure S21.** Staining images of the tumor dissections.	**S32**
**Figure S22.** The original images of western blot membranes.	**S33**
**Figure S23**. MALDI-TOF MS analysis of LSP1	**S34**
**Figure S24**. MALDI-TOF MS analysis of LSP2	**S35**
**Figure S25**. MALDI-TOF MS analysis of LSP3	**S36**
**Figure S26**. MALDI-TOF MS analysis of LSP4	**S37**
**Figure S27**. MALDI-TOF MS analysis of LSP5	**S38**
**Figure S28**. MALDI-TOF MS analysis of LSP6	**S39**
**Figure S29**. MALDI-TOF MS analysis of LSP7	**S40**
**Figure S30**. MALDI-TOF MS analysis of PROTAC-DBCO.	**S41**

**References**	**S42**

Detailed experimental procedures

Materials and Instruments. Unless otherwise noted, all reagents were used without further purification. Fmoc-protected amino acids, dibenzocyclooctyne-acid (DBCO acid), coupling reagents, and Rink amide resins were obtained from GL Biochem Ltd. (Shanghai, China). Trifluoroacetic acid and triisopropylsilane were purchased from J&K Scientific Ltd. (Beijing, China). Azide PEG3 N-hydroxysuccinimide (NHS) ester was purchased from Leyan (Shanghai, China). Trastuzumab (Catalogue number HY-P9907) and cetuximab (Catalogue number HY-P9905) were purchased from MedChemExpress (NJ, USA). Peptide characterization and purification were performed in RP-HPLC (Shimadzu, DGU-20A5, Japan). Peptide analysis was performed in an AutoFlex Speed LRF MALDI-TOF mass spectrometer (Bruker Daltonics, Germany). All gel images and Western blots were captured by a Bio-Rad ChemiDoc Image System (USA).

Peptide synthesis. All lysosome sorting sequence (LSS) peptides were synthesized based on manual Fmoc-SPPS chemistry. Briefly, Rink amide resins (GL Biochem Ltd.) with a loading capacity of 0.75 mmol/g were first swelled by DCM/DMF (50% v/v). For each coupling procedure, a 5-fold excess of protected amino acid, as well as the DBCO acid, HBTU, HOBt, and DIEA (with a ratio of 1: 1: 1: 2) in DMF, was added to the resin for 35 minutes with shaking at room temperature. The deprotection reaction of the Fmoc group was performed in 20% piperidine in DMF (v/v) after the resins were washed with DMF 5 times. After completion of the synthesis, the resin was washed thoroughly with DCM and DMF, then with methanol, and dried under vacuum. Normally, peptides were cleaved from the resin and side-chain deprotected by treatment with TFA / H_2O / TIPS (95/2.5/2.5) for 2h at room

temperature. The resin was then filtered and rinsed twice with TFA. The crude peptide was obtained by precipitation with cold diethyl ether.

Peptide purification and characterization. The crude peptide was dissolved in 50% ACN: 50% H_2O containing 0.1% TFA. After being filtered through a 0.2 μm filter, the peptide solution was injected into an RP-HPLC system (Shimadzu DGU-20A5, Japan) equipped with a C18 column (ZORBAX StableBond 300 C18, 9.4 x 250 mm, 5 μm). 0.1% TFA in H_2O (v/v) and 0.1% TFA in ACN (v/v) were used as the mobile phase A and B, respectively. For all analytical HPLC trials, the total flow rate was set to 1 mL/min, and the B concentration was increased from 5% to 95% over 13 min using a linear gradient. For the purification of peptides in a larger scale by semi-prep HPLC columns (ZORBAX StableBond 300 C18, 9.4 x 250 mm, 5 μm), the total flow rate was set to be 3 mL/min (gradient: 0-5 minutes 5% B, 5-30 minutes 5-65% B, 30-33 minutes 65-95% B, 33-36 minutes 95% B). The peptide peaks were collected, lyophilized, and confirmed by MALDI-TOF mass spectrometry analysis (Bruker Daltonics, Germany).

Cell culture. SK-BR-3 cells were purchased from Pricella (Wuhan, China). The cells were cultured in a humidified atmosphere (37 °C, 5% CO_2) in SK-BR-3 culture medium (Pricella, Catalogue number CM-0211).

Cytotoxicity studies. For the cytotoxicity measurement by Cell Counting Kit-8 (CCK8), 5000 SKBR-3 cells were seeded in a 96-well plate (Wuxi NEST Biotechnology Co., Ltd), and pre-incubated for 24 hours in a humidified incubator (at 37 °C, 5% CO_2). Added LSP coacervates at different concentrations

to the cells after washing the cells twice with PBS. Then, cells were incubated for 16 h. After washing the cells with PBS three times, 100 μL of culture medium containing 10 μL of CCK8 solution was added to each well. Special care was taken to avoid bubbles in the wells since they interfere with the O.D. reading. After 1 hour in the incubator, the absorbance at 450 nm was measured using a microplate reader.

Synthesis of BRD4 PROTAC

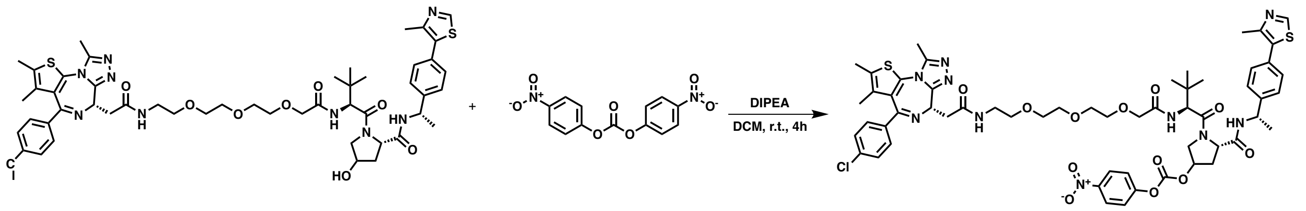

To a solution of BRD4 PROTAC (10.16 mg, 0.01 mmol) in DCM (1 mL), bis(4-nitrophenyl) carbonate (9.12 mg, 0.03 mmol) and DIPEA (5.22 μL, 0.03 mmol) were added, and the mixture was stirred at rt for 4 h. Then the solution was concentrated in vacuo. The residue was purified by flash column chromatography on silica gel (MeOH: DCM = 1:10) to afford PROTAC-PBA.

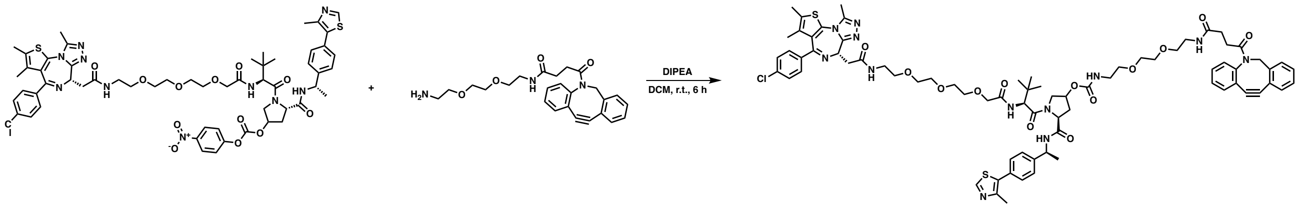

To a solution of 2 (10 mg, 0.008 mmol) in DCM (500 μL) was added DBCO PEG2 amine (10.4 mg, 0.024 mmol) and DIPEA (5.22 μL, 0.03 mmol), and the mixture was stirred at rt for 6 h. The solution was then concentrated in vacuo. The residue was purified by flash column chromatography on silica gel (MeOH: DCM = 1:10) to afford PROTAC-DBCO. To a 5 μM PROTAC-DBCO PBS solution, 2 μM (Tetramethylrhodamine 5-Carboxamido-(6-Azidohexanyl)), 5-isomer (TAMRA-N_3) was added to form the partially TAMRA-labeled PROTAC mixture for cell experiments without further purification.

PROTAC degradation. 5× 10^5 SK-BR-3 cells were seeded in a 12-well plate (Wuxi NEST Biotechnology Co., Ltd) and incubated for 24 hours in a humidified incubator (at 37 °C, 5% CO_2). 500 μL PROTAC BRD4 Degrader-5 (MCE) with or without **LSP-Coa** (1 mg/mL) was added to the cells, and the cells were incubated for 12 h. After washing the cells with PBS three times, 50 μL of radio-immunoprecipitation assay (RIPA) lysis buffer (MCE) was added to each well. After lysis, the samples were centrifuged at 14,000 g for 5 minutes at 4°C. The supernatants were then transferred to a new tube, and the protein concentration was determined using the BCA assay (Thermo Scientific) before Western blot analysis.

Western blotting. Primary antibodies: HER2/ErbB2 rabbit pAb (ABclonal, catalog number: A21768); EGFR mouse mAb (Proteintech, catalog number: 66455-1-Ig); BRD4 rabbit mAb (ABclonal, catalog number: A12677); GAPDH rabbit pAb (ABclonal, catalog number: AC001). Secondary antibodies: HRP-conjugated goat anti-rabbit IgG(H+L) (Proteintech, catalog number: SA00001-2); HRP-conjugated horse anti-mouse IgG(H+L) (Cell Signaling Technology, catalog number: 7076S). 10 μg of lysate was separated by SDS–PAGE (10% gel) and then transferred to a polyvinylidene fluoride (PVDF) membrane. After transfer, blocked in TBST (10 mM Tris–HCl (pH 8), 150 mM NaCl, 0.05% Tween 20) with 5% non-fat milk for 1 hour at room temperature with gentle shaking. Membranes were incubated with primary antibodies at 4 °C with gentle shaking overnight, then washed three times for 5 minutes each in TBST. The membrane was incubated with the secondary antibody in TBST for 1 h.

General protocol for immunofluorescent labeling. After adding **LSP-Coa**, cells were washed with PBS for 3 times. Subsequently, the cells were fixed in 4% paraformaldehyde in PBS (pH 7.4) for 15

minutes at room temperature. The cells were washed three times with PBS. Then, we incubated the cells for 10 min with PBS containing 0.2% Triton X-100. After extensive washing, we incubated cells with 1% BSA in PBST (PBS + 0.1% Tween 20) for 1 hour to block nonspecific binding. Next, the cells were incubated with the primary antibody in 1% BSA overnight at 4℃. Finally, the cells were incubated with fluorescent anti-rabbit secondary antibody in 1% BSA for 3 hours at room temperature in the dark, after washing away excess primary antibody. The fluorescent signals were observed using a confocal microscope. Primary antibodies: HER2/ErbB2 rabbit pAb (ABclonal, catalog number: A21768); RAB5A Rabbit pAb (ABclonal, catalog number: A1180); RAB7A pAb (Proteintech, catalog number: 55469-1-AP); fluorescent anti-rabbit secondary antibody: ABflo® 647-conjugated Goat anti-Rabbit IgG (H+L) (ABclonal, catalog number: AS060).

Appendix. A figure containing the uncropped Western blot images is shown in Figure S22. Detailed characterizations of the peptides and compounds are shown in Figures S23-S30.

Table S1. Screening of peptide derivatives for coacervate states.

Name	Sequence	Condensate states
LSP-1	DBCO-PEG2-NPGY-amide	Coacervate
LSP-2	DBCO-NPGY-PEG2-NPGY-amide	Coacervate
LSP-3	DBCO-NPGY-acid	Solution
LSP-4	DBCO-NPGY-amide	Gel-like particles
LSP-5	DBCO-L-PEG2-NPGY-amide	Coacervate (unstable)
LSP-6	DBCO-F-PEG2-NPGY-amide	Coacervate (unstable)
LSP-7	DBCO-GG-NPGY-amide	Gel-like particles

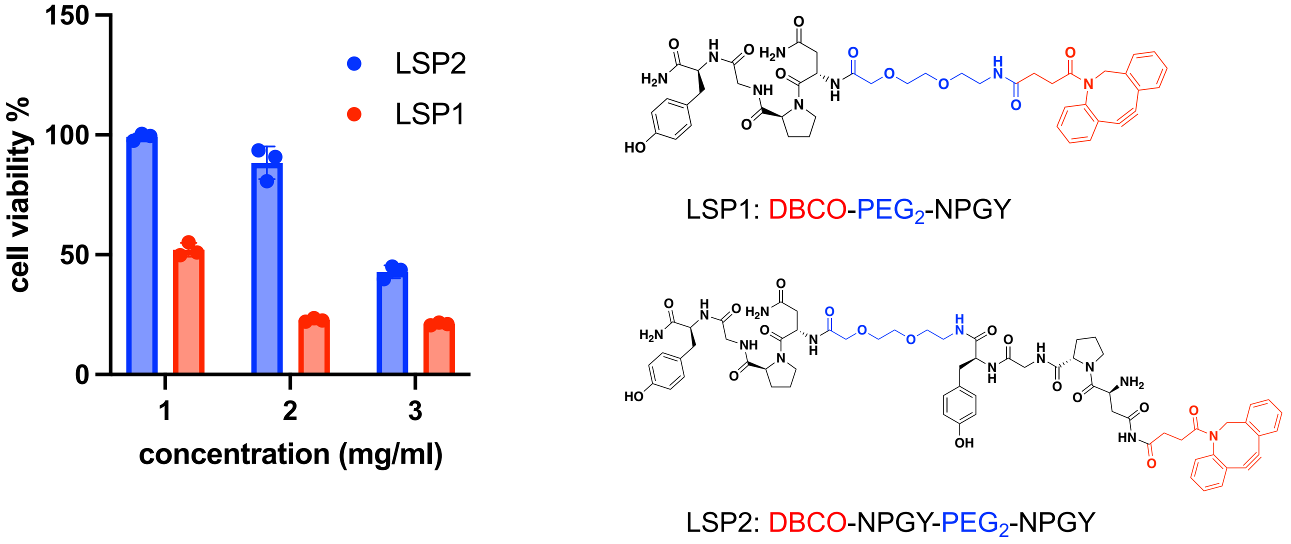

Figure S1. Cytotoxicity of LSP1 and LSP2. Peptides at varying concentrations were incubated with SK-BR-3 cells for 16 hours, and the CCK-8 assay was used to assess cell viability.

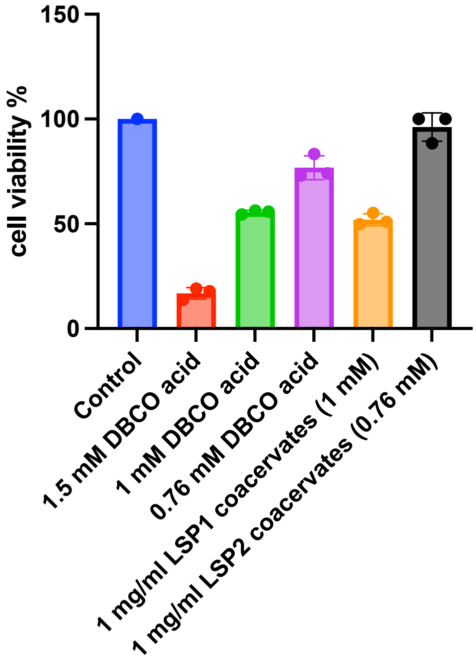

Figure S2. Cytotoxicity of DBCO-containing compounds. Peptides at varying concentrations were incubated with SK-BR-3 cells for 16 hours, and cell viability was assessed using the CCK-8 assay.

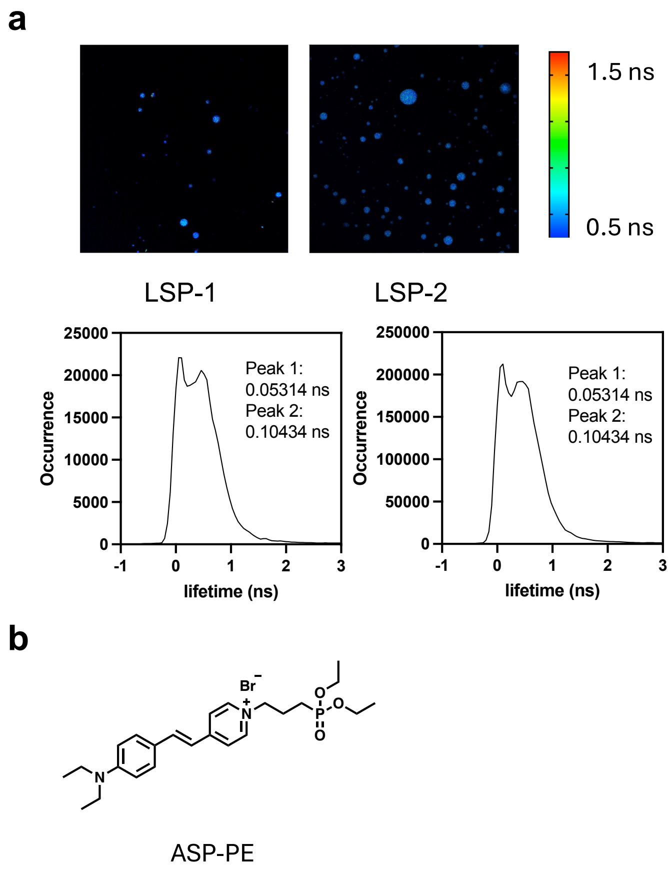

Figure S3. Qualitative evaluation of the fluidity of peptide coacervates. (a) Images of LSP-1 and LSP-2 coacervates showing the fluorescence lifetime of ASP-PE in the droplets. (b) Chemical structure of the fluorescent probe ASP-PE. [1]

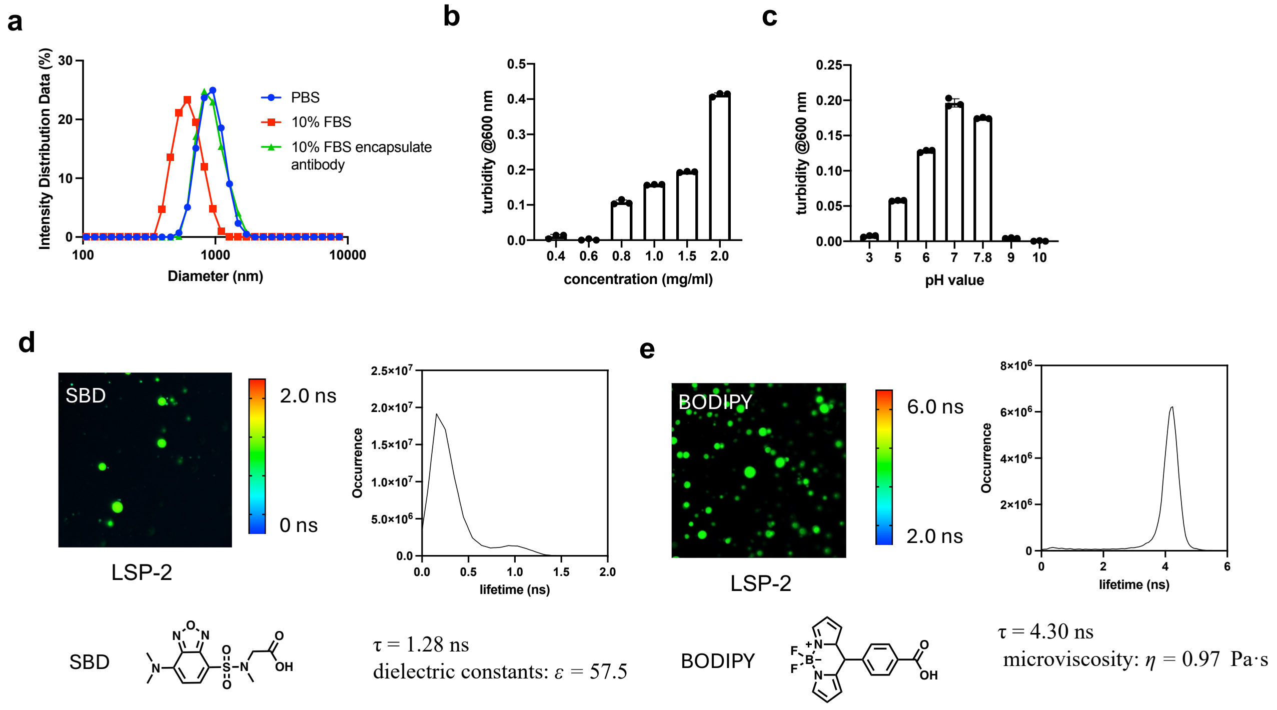

Figure S4. Physical properties of **LSP-Coa**. (a) Coacervate sizes measured by dynamic light scattering (DLS) at 1 mg/mL in PBS buffer (pH 7.4). (b) Turbidity of the **LSP-Coa** preparations at different concentrations in PBS buffer (pH 7.4). (c) Turbidity of **LSP-Coa** (1 mg/ml) in solutions of different pH values. (d) Fluorescence lifetime images of **LSP-Coa** droplets using SBD as the probe to estimate the dielectric constant. (e) Fluorescence lifetime images of **LSP-Coa** droplets using BODIPY as the probe to estimate the microviscosity. [2]

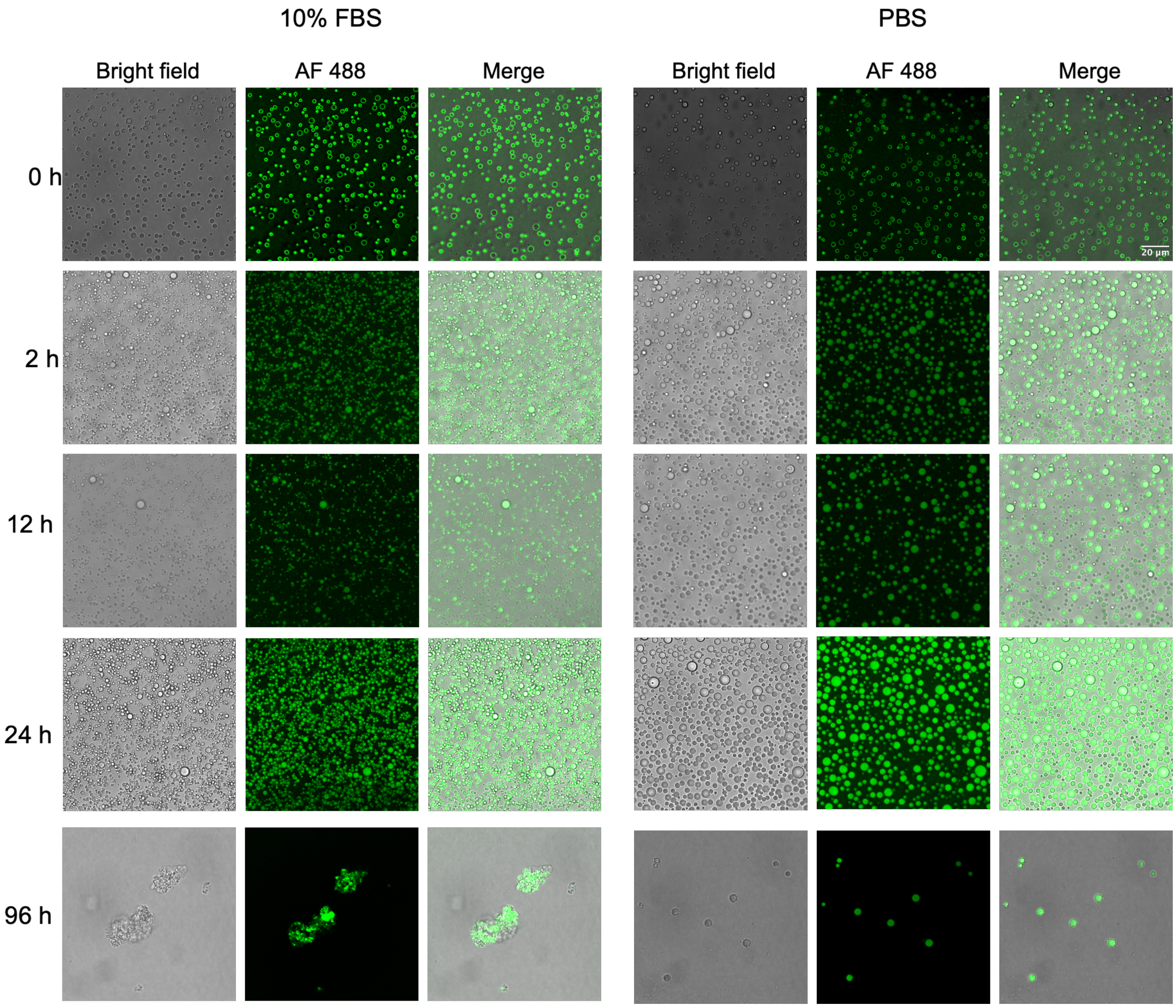

Figure S5. Confocal fluorescent images of **LSP-Coa** maintained in PBS or 10% FBS for 96 hours. The AlexaFluo 488 signal was shown to indicate the integrity of the droplets.

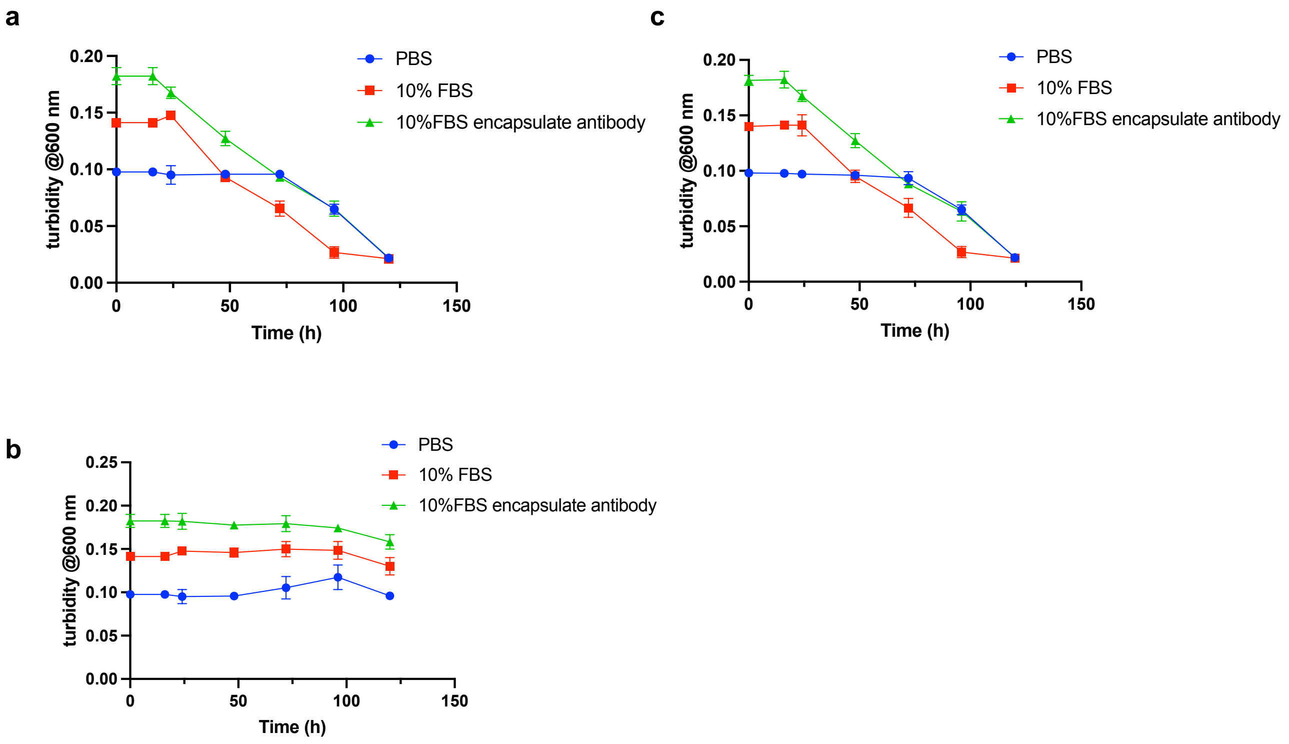

Figure S6. Stability of **LSP-Coa** under different conditions. Turbidity of **LSP-Coa** in PBS, 10% FBS, and 10% FBS with the antibody encapsulated at (a) 37℃, (b) 4 ℃, and (c) in the presence of 0.01 mg/mL PEG2000.

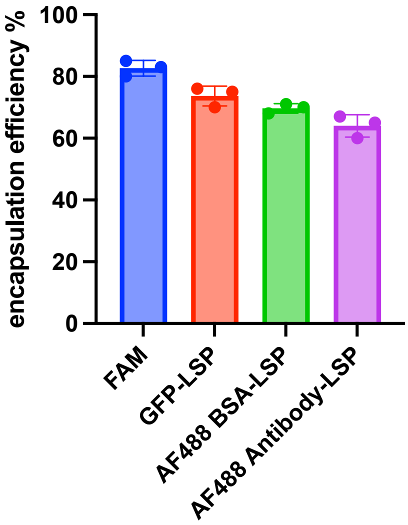

Figure S7. Encapsulation of different fluorescent proteins or fluorescent dyes in the **LSP-Coa**. Briefly, the fluorescence signal of the cargoes at 0.01 mg/mL was measured as F0. LSP was added to the protein solution to form **LSP-Coa,** with a final molecular concentration of 1 mg/mL LSP peptide and 0.01 mg/mL cargo. The solution was then centrifuged, and the fluorescence signal of the supernatant was measured as F1. The encapsulation efficiency was calculated as (F0-F1)/F0×100%. Data are presented as the mean ± s.d. of n = 3 independent experiments.

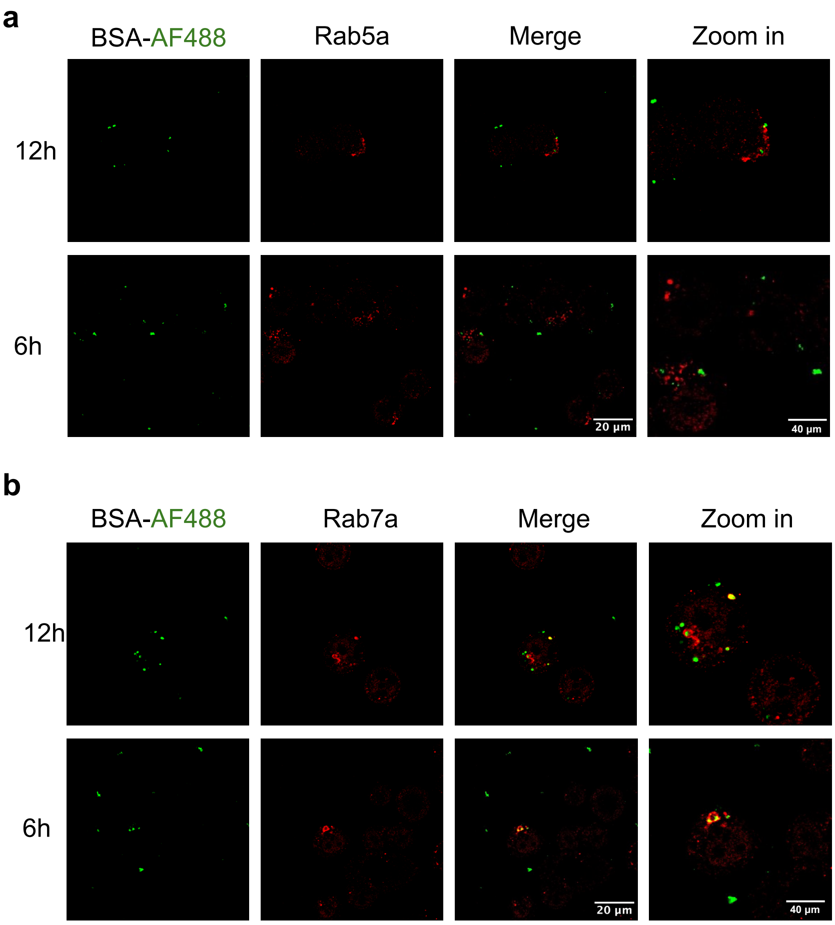

Figure S8. Confocal fluorescent images showing the co-localization of **LSP-Coa**/protein complex with late endosomes. (a) Confocal fluorescent images showing the lack of co-localization of Rab5a (early endosome marker) and AF 488-labeled BSA delivered by **LSP-Coa**. (b) Confocal fluorescent images showing co-localization of Rab7a (late endosome marker) and AF 488-labeled BSA delivered by **LSP-Coa**. Green: AF 488-labeled BSA encapsulated in **LSP-Coa**. Red: (a) Rab5a, or (b) Rab7a.

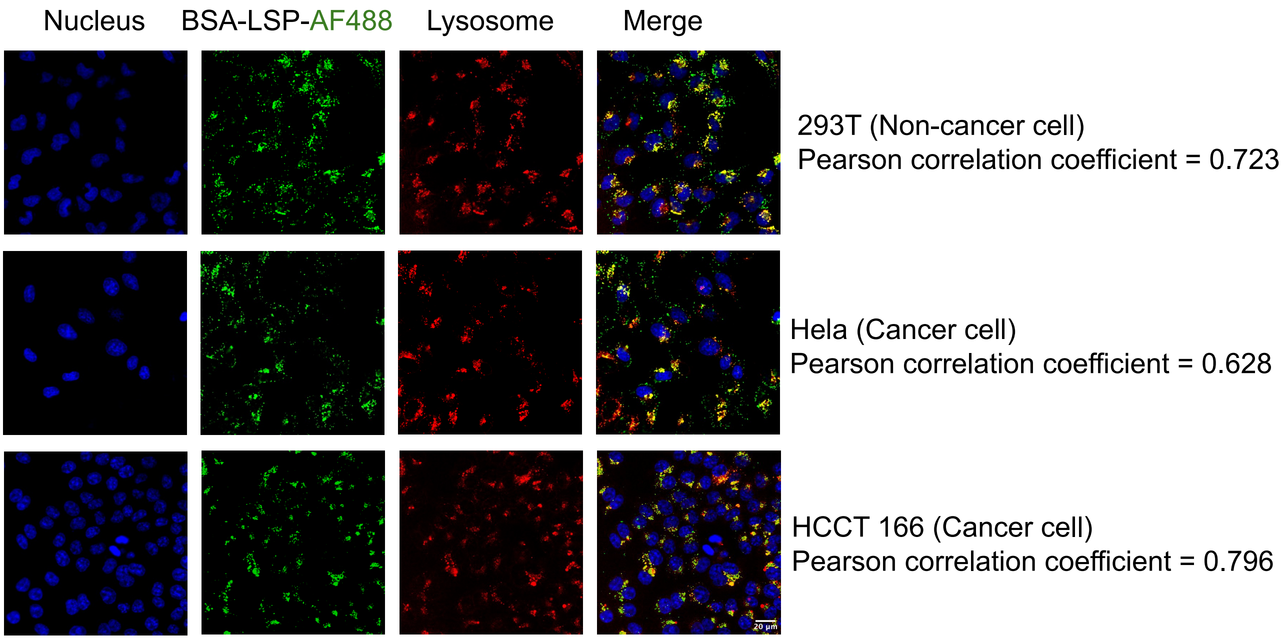

Figure S9. Protein delivery to different cell lines using **LSP-Coa**. **LSP-Coa** can deliver protein cargoes to cancer cell lines (HCCT 166 and Hela) and Non-cancer cell lines (293T). Blue: Hoechst; Green: AF 488-BSA-LSP; Red: LysoTracker Deep Red. Pearson correlation coefficient values are calculated by Image J JACOP.

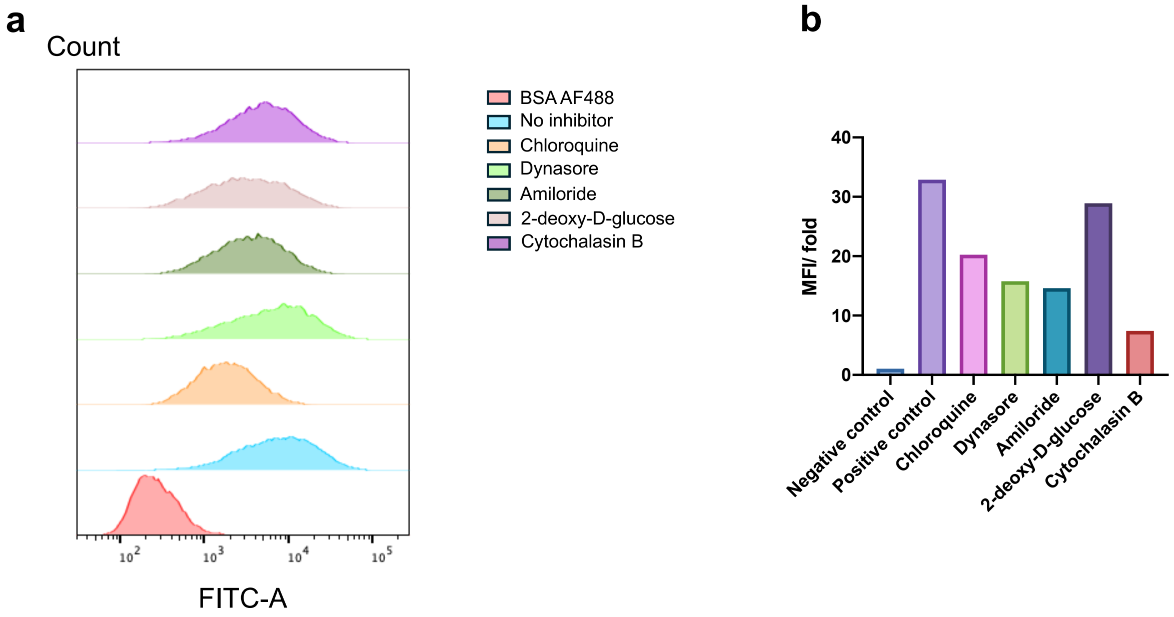

c

Group	Negative control	Positive control	Chloroquine	Dynasore	Amiloride	2-deoxy-D-glucose	Cytochalasin B
MFI/ Fold	1	33	20	16	15	29	7
Pathways affected			CME	CME and FEME	Macro	energey dependent	Macro and phago

Figure S10. Flow cytometry data showing inhibition of **LSP-Coa** internalization by endocytosis inhibitors. (a) Flow cytometry results of the BSA-AF488 internalization with different inhibitors. (b) Quantification data comparing the mean values of the fluorescent intensity. (c) Summarization showing the pathways affected by inhibitors. CME, clathrin- and dynamin-dependent; FEME, clathrin-independent but dynamin-dependent; Macro, macropinocytosis; Phago: phagocytosis.

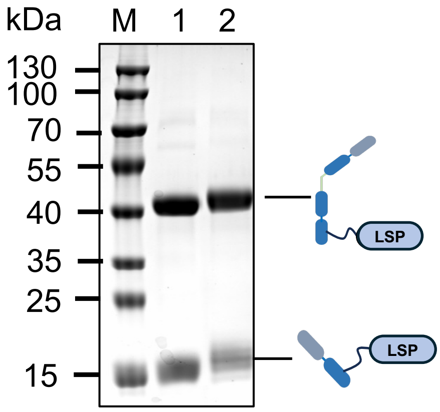

Figure S11. LSP-antibody conjugation. The SDS-PAGE showed that the LSP peptide was covalently conjugated to the antibody. Lane M: Protein marker, lane 1: Tras-N_3, lane 2: Tras-LSP. Tras: Trastuzumab.

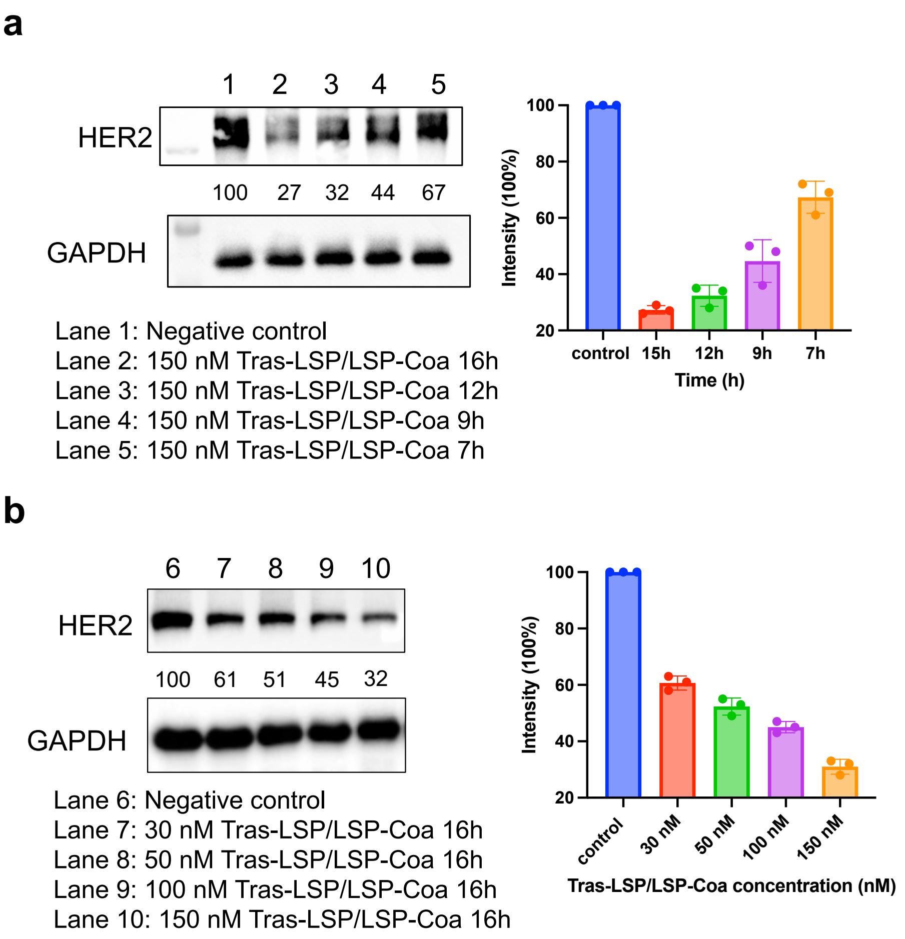

Figure S12. Dose- and time-dependent **LSP-Coa**-mediated degradation of cell-surface antigens. (a). HER2 western blot (left) of SK-BR-3 cells treated with 150nM Tras-LSP/**LSP-Coa** for 16, 12, 9, and 7 hours; and quantitative analysis of the western blot (right) (b). HER2 western blot (left) of SK-BR-3 cells treated with different concentrations of Tras-LSP/**LSP-Coa** for 16 hours; and quantitative analysis of the western blot (right) HER2 levels were normalized using GAPDH as the control: relative HER2 % = (HER2 $signal_{expt}$ / GAPDH $signal_{expt}$)/ (HER2 $signal_{no\ treatment}$ / GAPDH $signal_{no\ treatment}$) × 100%. Data are presented as the mean ± s.d. of n = 3 independent experiments.

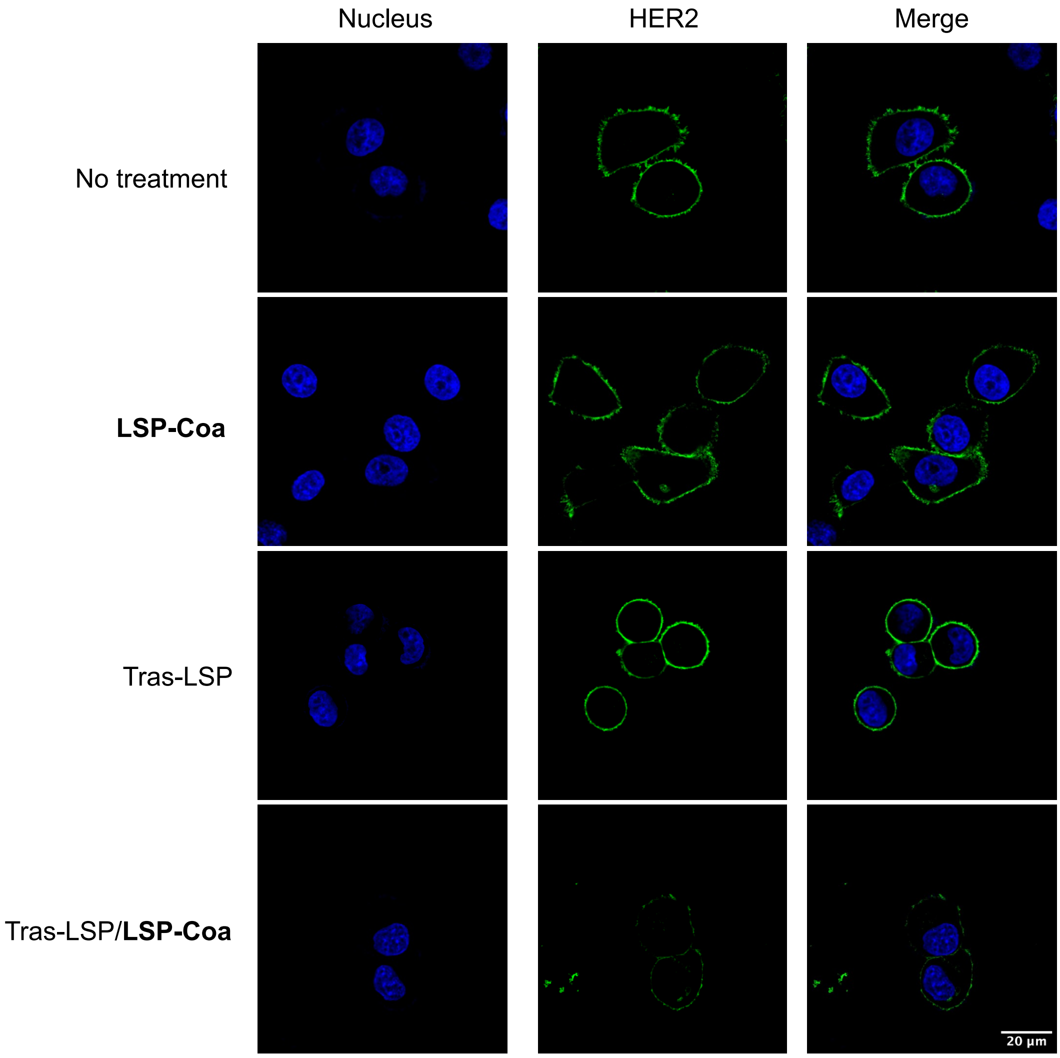

Figure S13. Immunofluorescent images showing down-regulation of HER2 on SK-BR-3 cells after different degradation through the confocal microscope. Primary antibody: HER2/ErbB2 rabbit pAb (ABclonal, catalog number: A21768); Green: AF 488 labeled anti-Rabbit antibody; Blue: DAPI.

a **Condition 1**: BSA-LSP-AF488/ BSA-LSP-Cy5/**LSP-Coa**, both proteins simultaneously loaded in **LSP-Coa**

BSA-LSP-AF488 BSA-LSP-Cy5 Merge Bright field

0 min

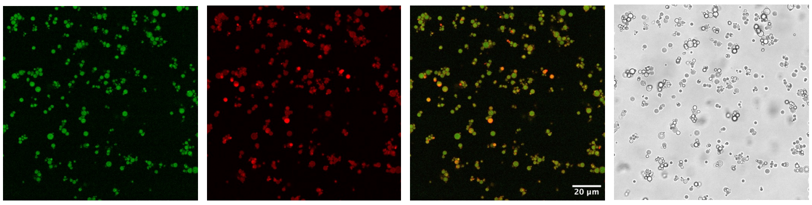

Pearson correlation coefficient: 0.708

Condition 2: mixture of BSA-LSP-AF488/**LSP-Coa** + BSA-LSP-Cy5/**LSP-Coa**

BSA-LSP-AF488 BSA-LSP-Cy5 Merge Bright field

0 min

10 min

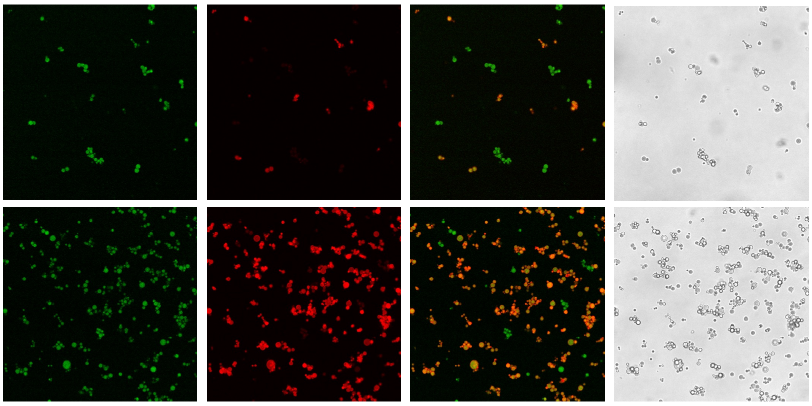

Pearson correlation coefficient: 0.346

Pearson correlation coefficient: 0.664

b **Condition 1**: Tras-LSP-AF488/Ctx-LSP-Cy5/**LSP-Coa**, both antibodies simultaneously loaded in **LSP-Coa**

Tras-LSP-AF488 Ctx-LSP-Cy5 Merge Bright field

0 min

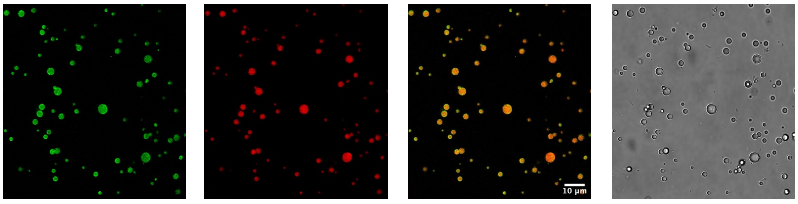

Pearson correlation coefficient: 0.654

c **Condition 1**: BSA-LSP-AF488/BSA-LSP-Cy5/**LSP-Coa**, then added to SK-BR3 cells for 12 hours

BSA-LSP-AF488 BSA-LSP-Cy5 Merge Bright field

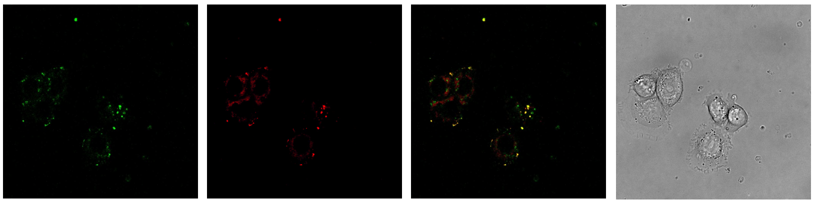

Pearson correlation coefficient: 0.696

Condition 2: mixture of BSA-LSP-AF488/**LSP-Coa** + BSA-LSP-Cy5/**LSP-Coa**, then added to SK-BR-3 cells for 12 hours

BSA-LSP-AF488 BSA-LSP-Cy5 Merge Bright field

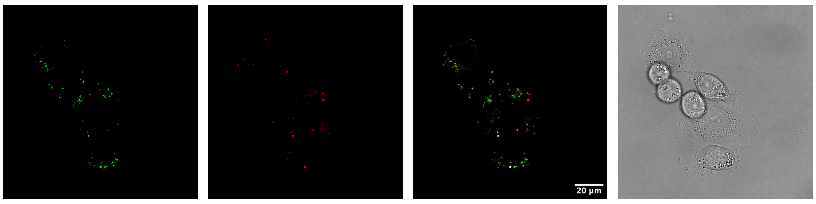

Pearson correlation coefficient: 0.246

Figure S14. Comparison of the two conditions *in vitro* and in cells. (a) Confocal fluorescent images of the distribution of two proteins using different encapsulation methods in PBS buffer. Green: AF488 labeled BSA-LSP conjugates; Red: Cy5 labeled BSA-LSP conjugates; Pearson correlation coefficient values are calculated by Image J JACOP. (b) Confocal fluorescent images revealed the encapsulation of two antibodies within **LSP-Coa**. Green: AF488 labeled Trastuzumab-LSP conjugates; Red: Cy5 labeled Cetuximab-LSP conjugates. (c) Confocal fluorescent images of the distribution of two proteins in SK-BR-3 cells using different encapsulation methods. Green: AF488 labeled BSA-LSP conjugates; Red: Cy5 labeled BSA-LSP conjugates; Pearson correlation coefficient values are calculated by Image J JACOP.

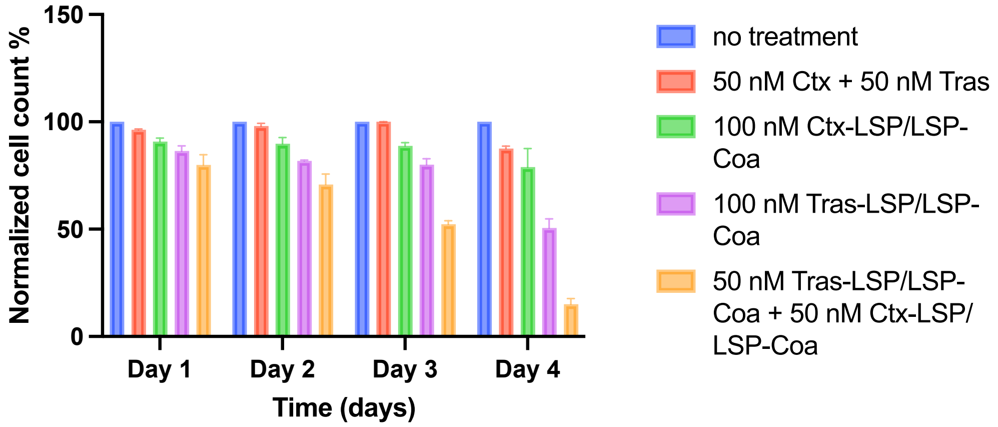

Figure S15. Inhibition of cell proliferation using antibody-LSP/**LSP-Coa**. The cells were treated with 100 nM Ctx-LSP/**LSP-Coa**, 100 nM Tras-LSP/**LSP-Coa**, 50 nM Tras-LSP/**LSP-Coa** + 50 nM Ctx-LSP/**LSP-Coa**, and 50 nM Ctx + 50 nM Tras as the control group on day 0 and day 2. The number of cells was tested daily using CCK8, and cell numbers were normalized using the no-treatment group as the control. The normalized cell number percentage was calculated as follows: Normalized cell number % = (cell numbers of each group/cell numbers of no treatment) × 100%. Data are presented as the mean ± s.d. of n = 3 independent experiments.

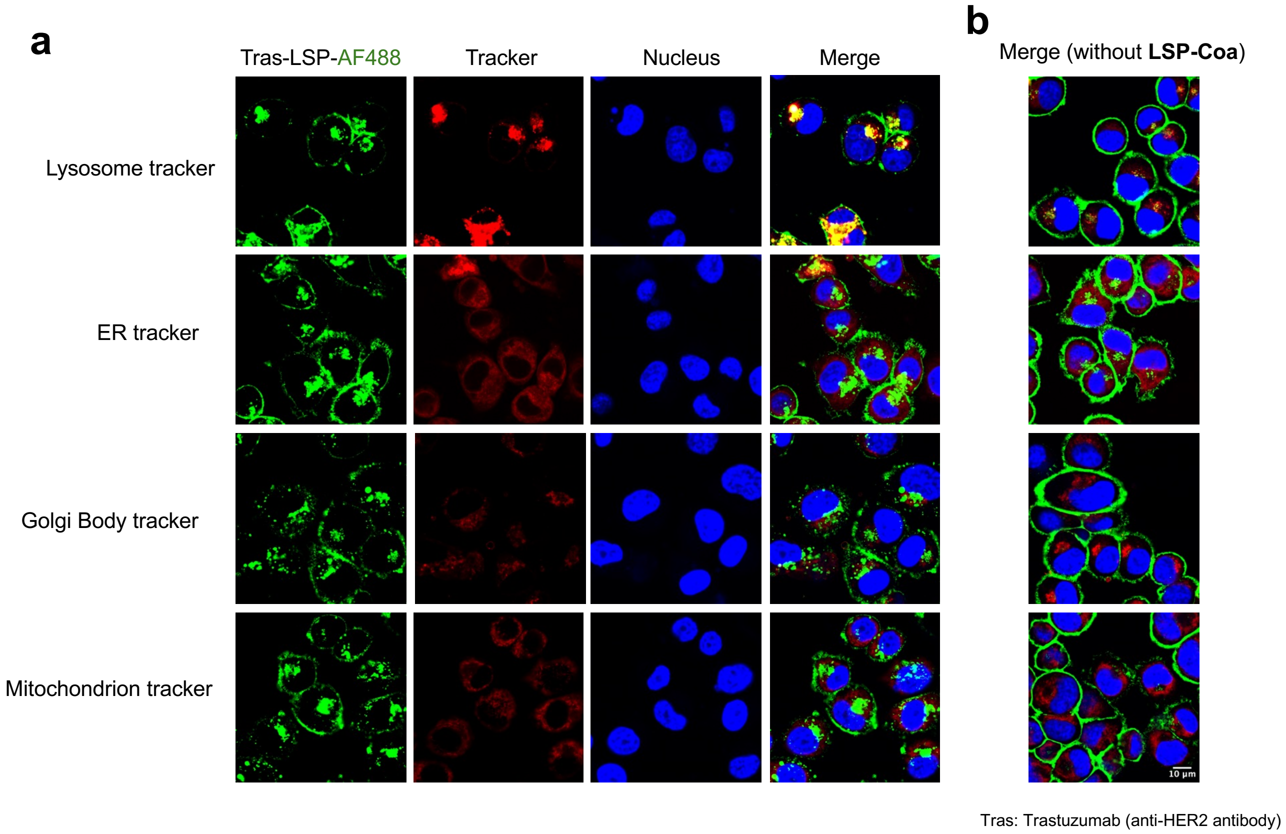

Figure S16. Confocal fluorescent images for co-localization of the antibody and different organelles. (a), (b) Confocal fluorescent images for co-localization of different organelles and AF 488-labeled Tras-LSP conjugate (a) with and (b) without LSP coacervates. Blue: Hoechst; Green: AF 488-labeled Tras-LSP conjugate; Red (up to down): Lyso-Tracker Deep Red, ER-Tracker Red, Golgi-Tracker Red, and Mito-Tracker Red.

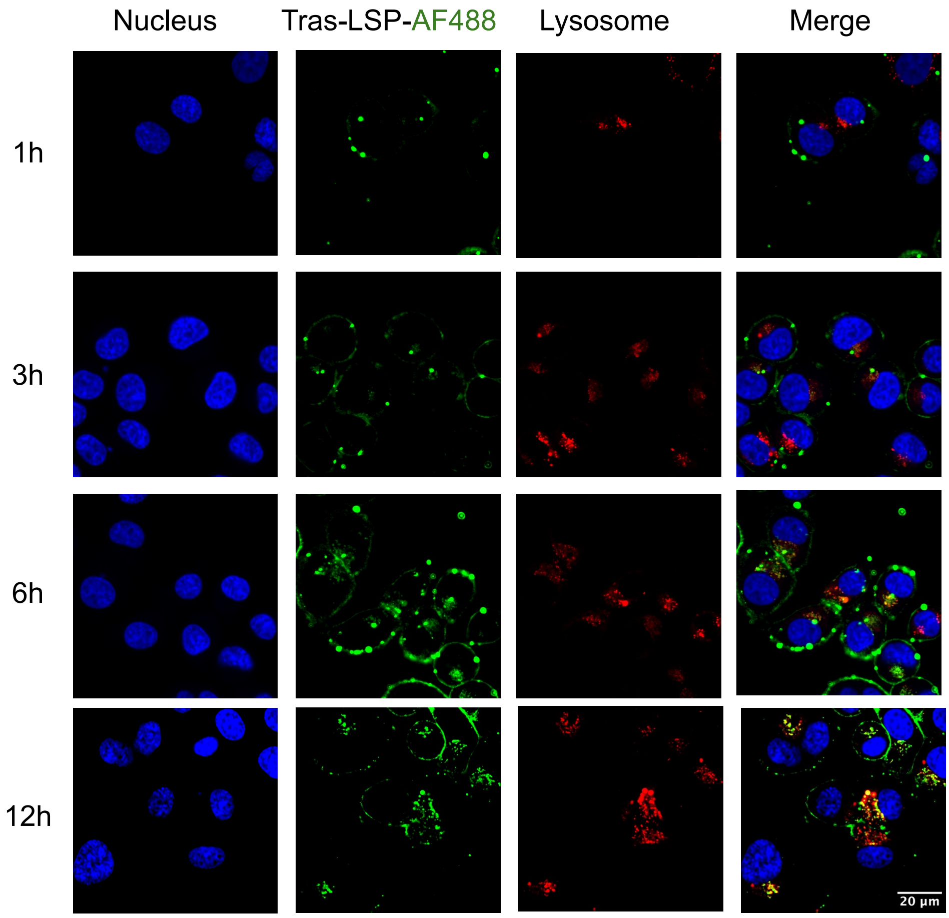

Figure S17. Confocal fluorescent images for Tras-LSP-AF488 delivery to SK-BR-3 cells through **LSP-Coa** with different incubation times. Blue: Hoechst; Green: AF 488-labeled Tras-LSP conjugate; Red: Lyso-Tracker Deep Red.

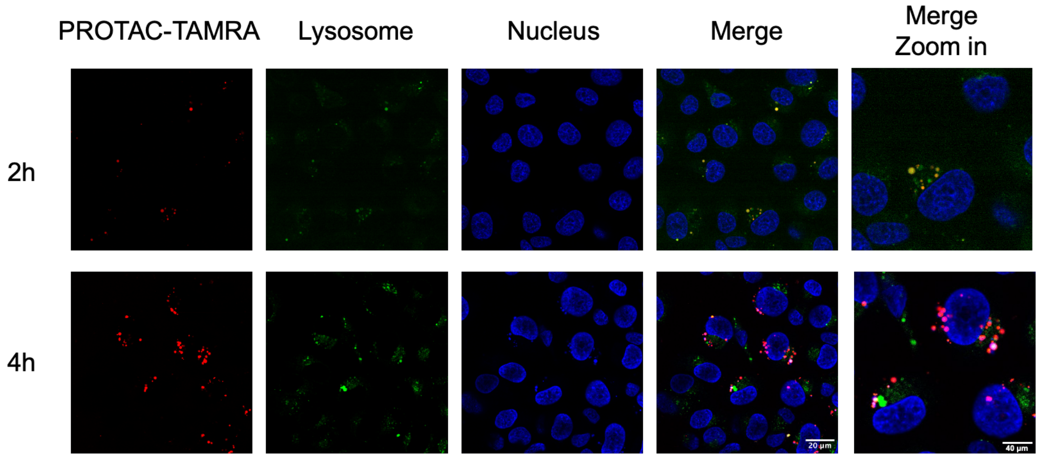

Figure S18. Confocal fluorescent images for PROTAC location in cells. (a) Confocal fluorescent images showed the location of BRD4 PROTAC-TAMRA (5(6)-Carboxytetramethylrhodamine) in cells with **LSP-Coa** containing PROTAC-TAMRA for 2 and 4 hours of incubation (Red: PROTAC-TAMRA; Green: Lyso-tracker Green; Blue: Hoechst).

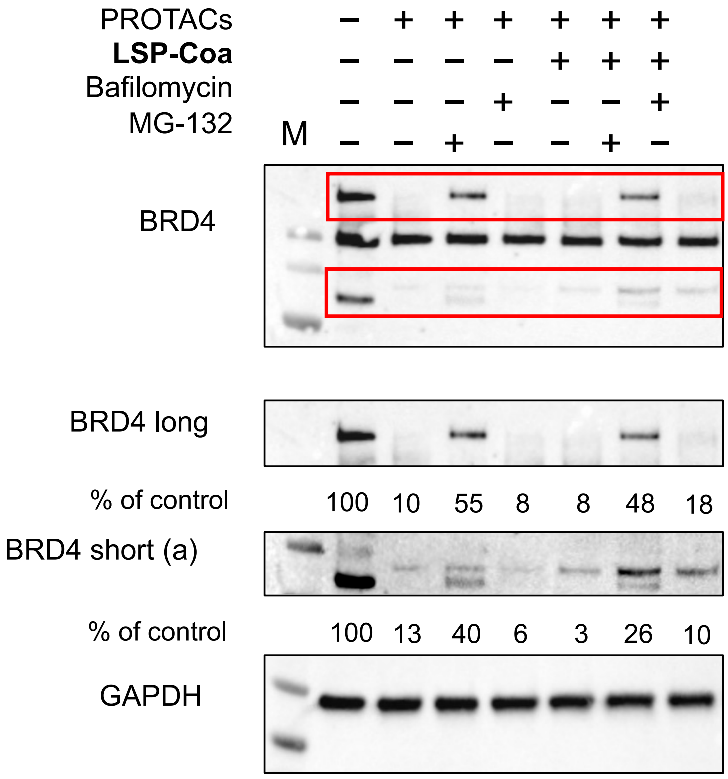

Figure S19. Mechanism of BRD4 degradation with or without **LSP-Coa**. The results showed that the degradation of BRD4 was significantly blocked by proteasome inhibitors but not lysosome inhibitors.

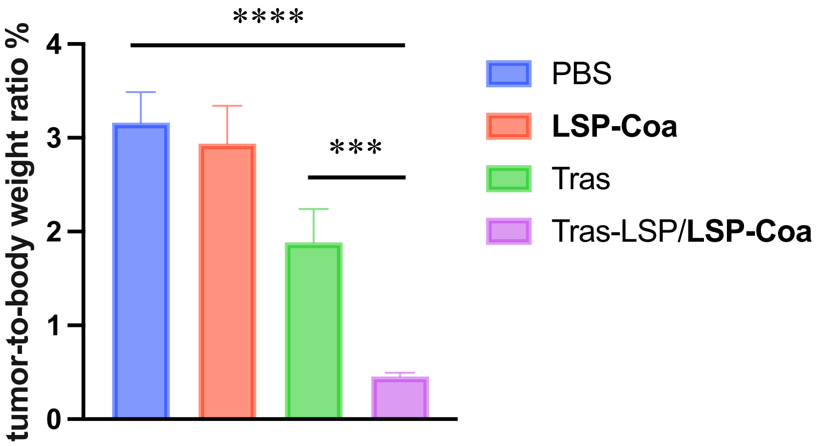

Figure S20. Tumor-to-body weight ratio for *in vivo* experiments. Analysis of the tumor-to-body weight ratio on day 14 between the control and experimental groups. Data are presented as the mean ± s.d. of n = 3 independent experiments. ***$P < 0.001$, ***$P < 0.0001$ according to two-sided Student's t-test.

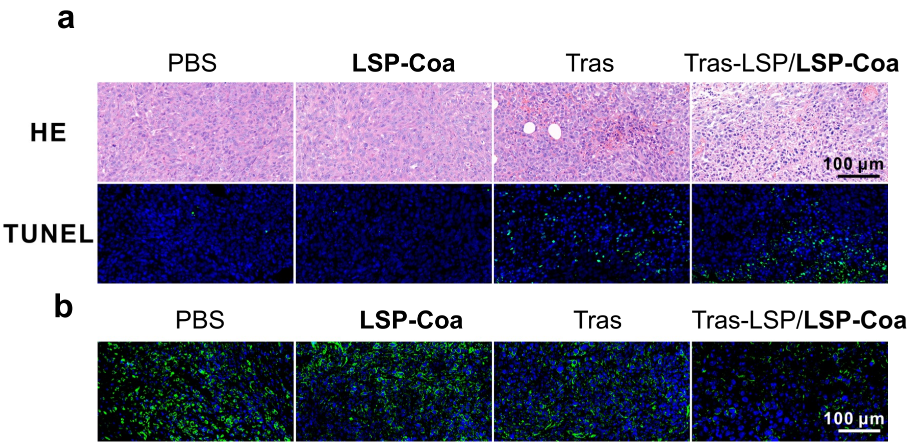

Figure S21. Staining images of the tumor dissections. (a) HE and TUNEL staining images of the tumor dissection 16 hours after injection. (b) The HER2 level of the tumor dissection 16 hours after injection.

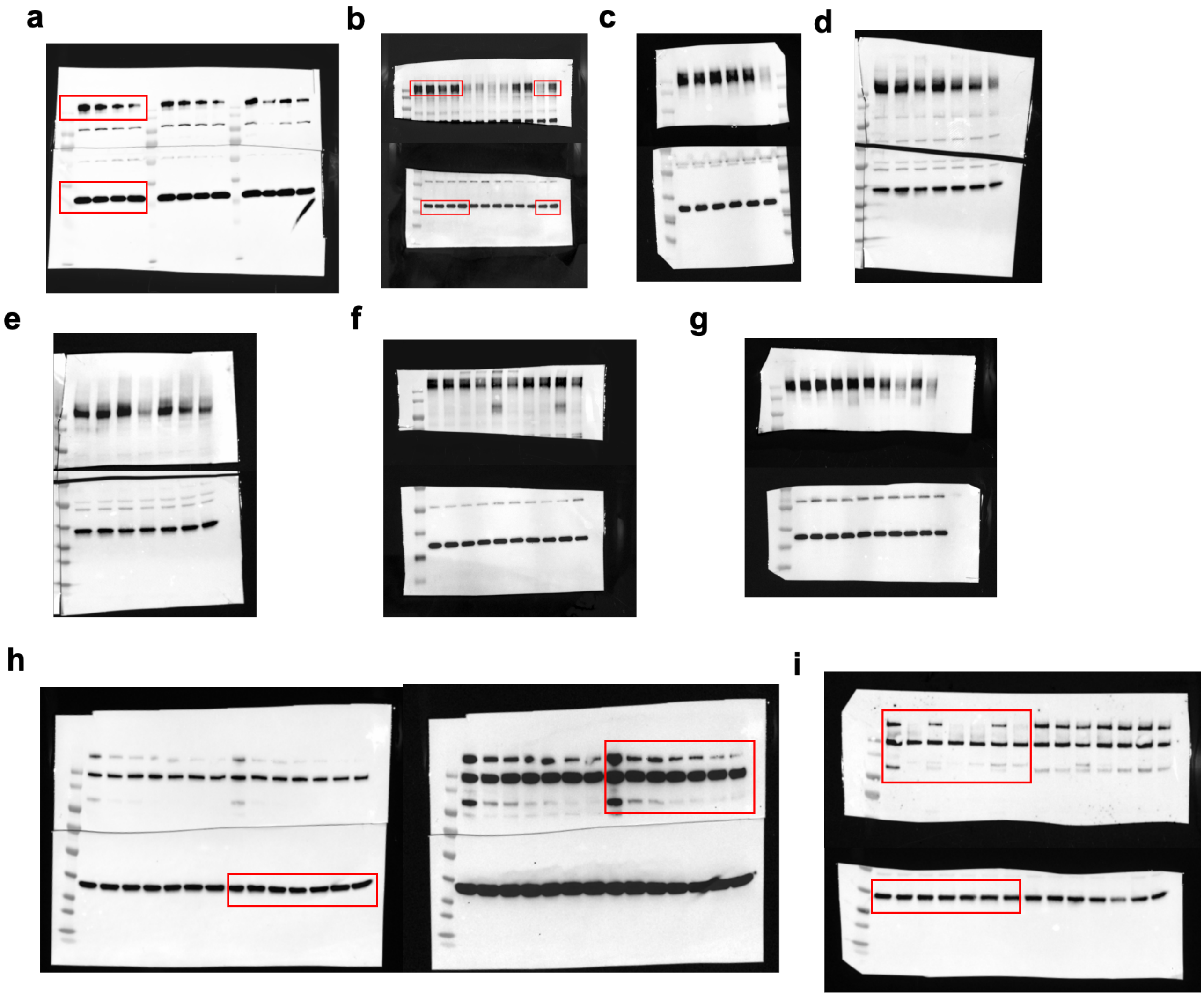

Figure S22. The original images of western blot membranes. (a) Figure 3b, (b) Figure 3d, (c) Figure 3e, (d) Figure 4b, (e) Figure 4c, (f) Figure 5b, (g) Figure 5c, (h) Figure 6b, (i) Figure S15.

LSP-1

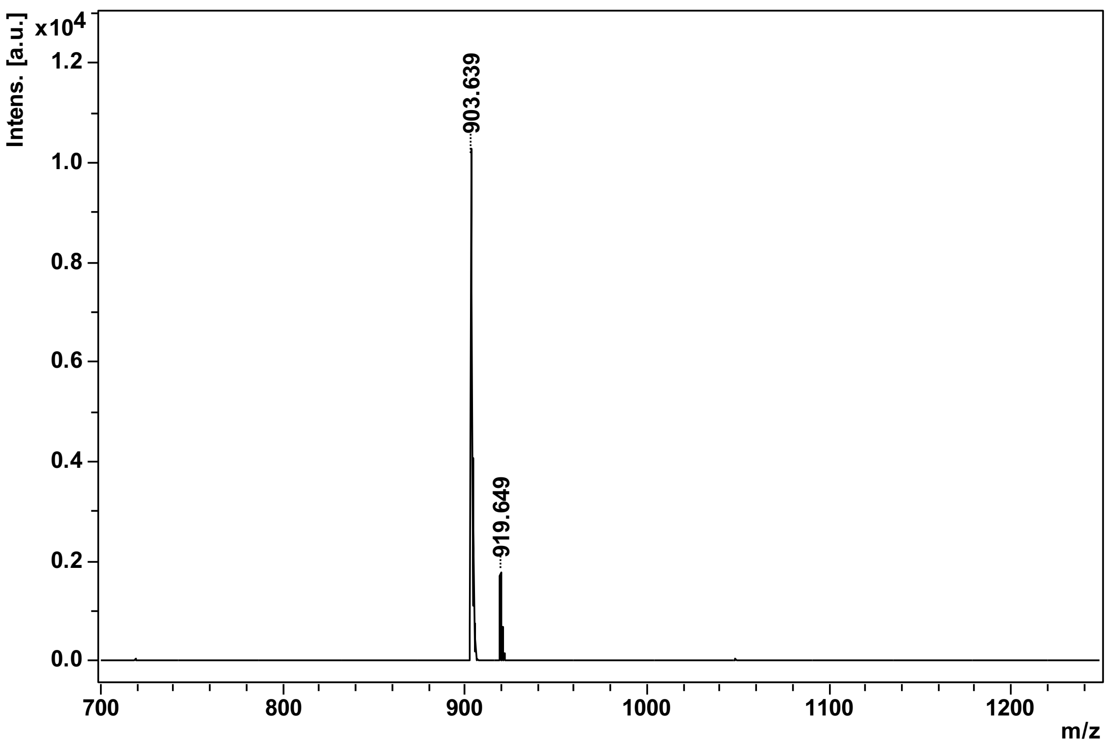

Figure S23. MALDI-TOF MS analysis of LSP1. HRMS calculated for $C_{45}H_{52}N_8O_{11}Na$ $[M+Na]^+$ 903.364; found 903.639. $C_{45}H_{52}N_8O_{11}K$ $[M+K]^+$ 919.338; found 919.649.

LSP-2

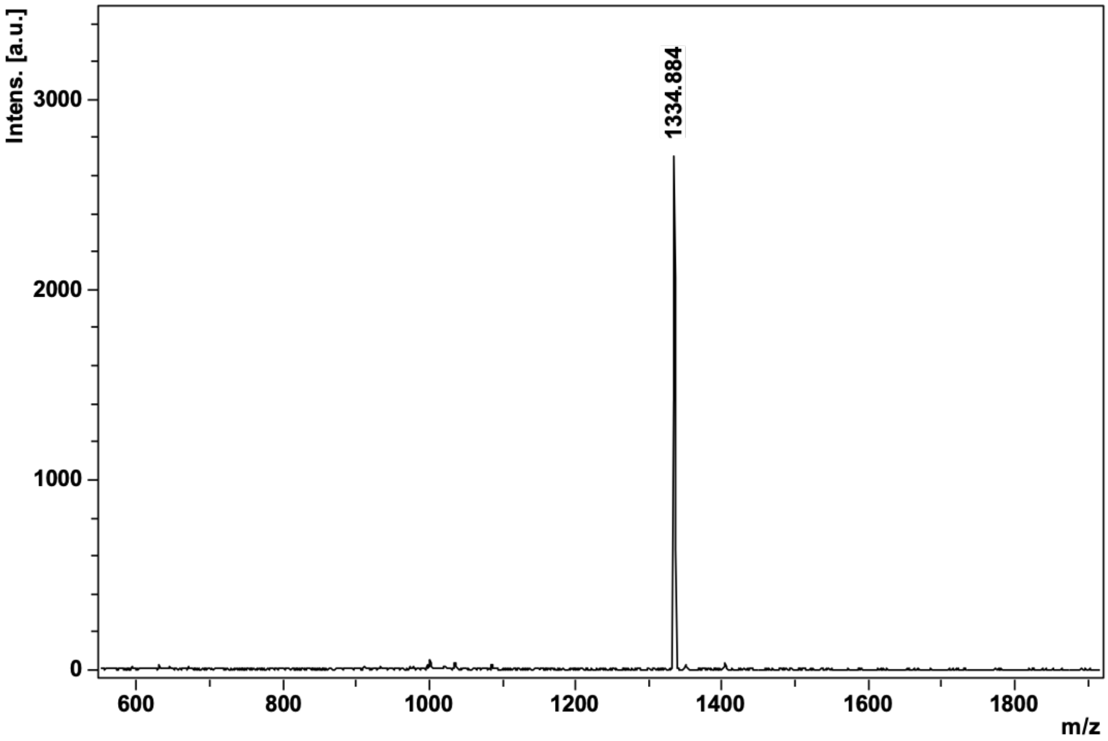

Figure S24. MALDI-TOF MS analysis of LSP2. HRMS calculated for $C_{65}H_{77}N_{13}O_{17}Na$ $[M+Na]^+$ 1334.545; found 1334.884.

LSP-3

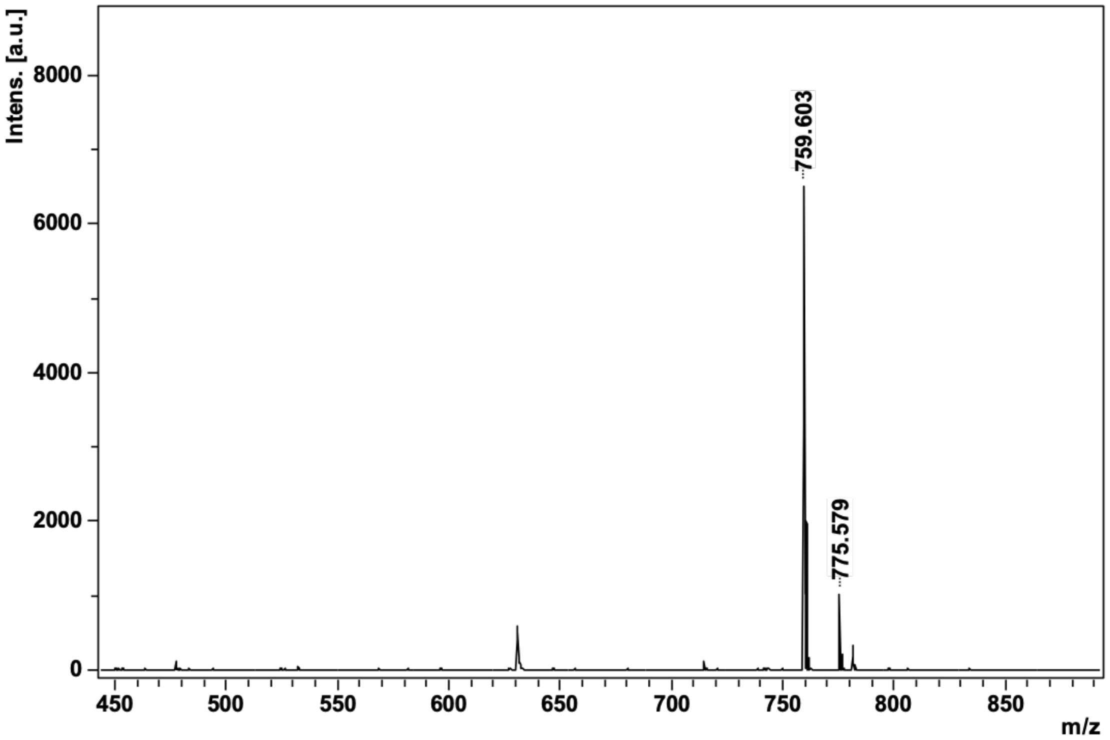

Figure S25. MALDI-TOF MS analysis of LSP3. HRMS calculated for $C_{39}H_{40}N_6O_9Na$ $[M+Na]^+$ 759.275; found 759.603. $C_{39}H_{40}N_6O_9K$ $[M+K]^+$ 775.249; found 775.579.

LSP-4

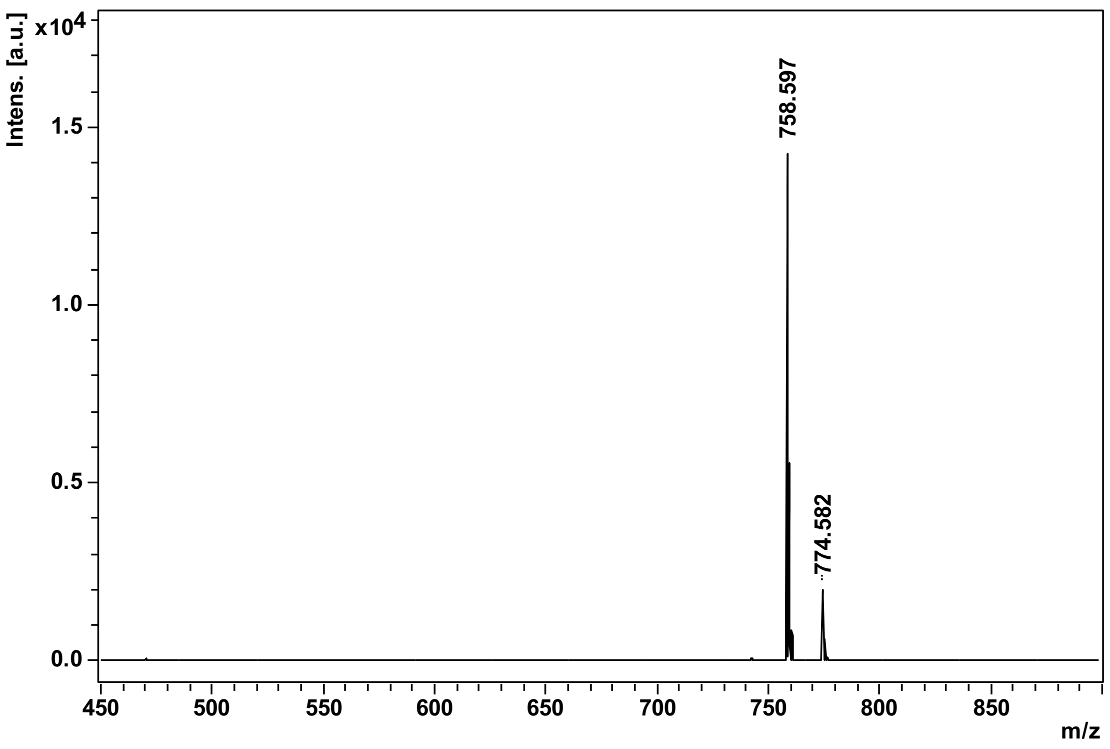

Figure S26. MALDI-TOF MS analysis of LSP4. HRMS calculated for $C_{39}H_{41}N_7O_8Na$ $[M+Na]^+$ 758.291; found 758.597. $C_{39}H_{41}N_7O_8K$ $[M+K]^+$ 774.265; found 774.582.

LSP-5

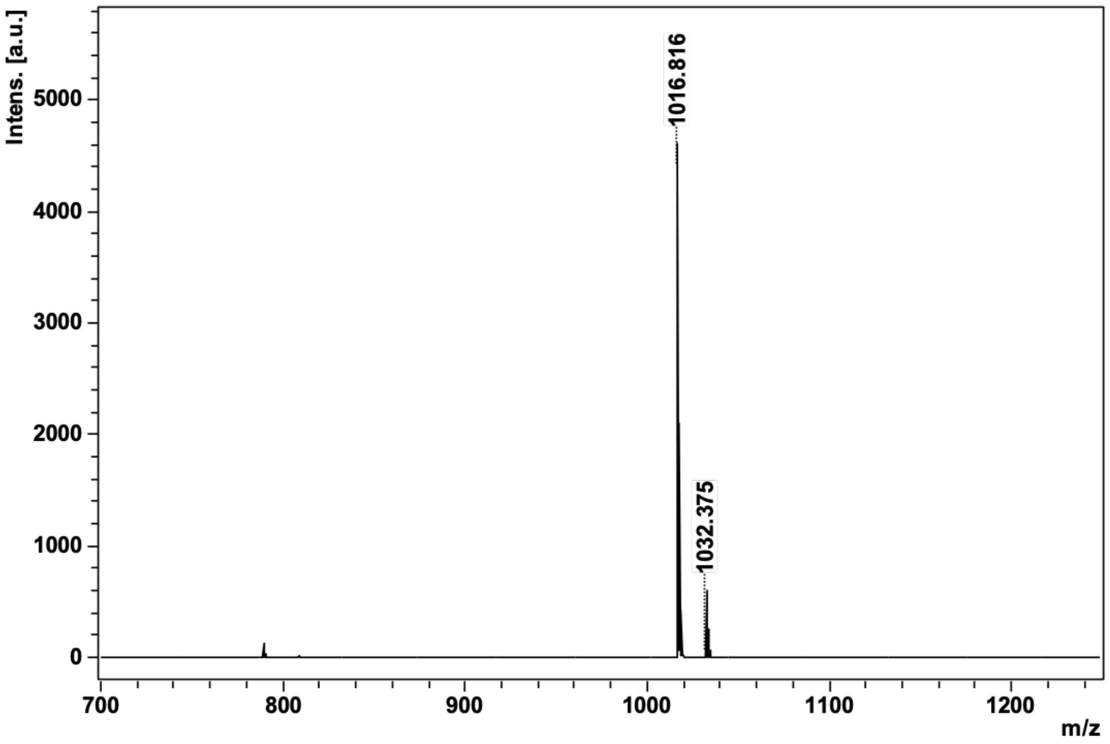

Figure S27. MALDI-TOF MS analysis of LSP5. HRMS calculated for $C_{51}H_{63}N_9O_{12}Na$ $[M+Na]^+$ 1016.449; found 1016.816. $C_{51}H_{63}N_9O_{12}K$ $[M+K]^+$ 1032.423; found 1032.375.

LSP-6

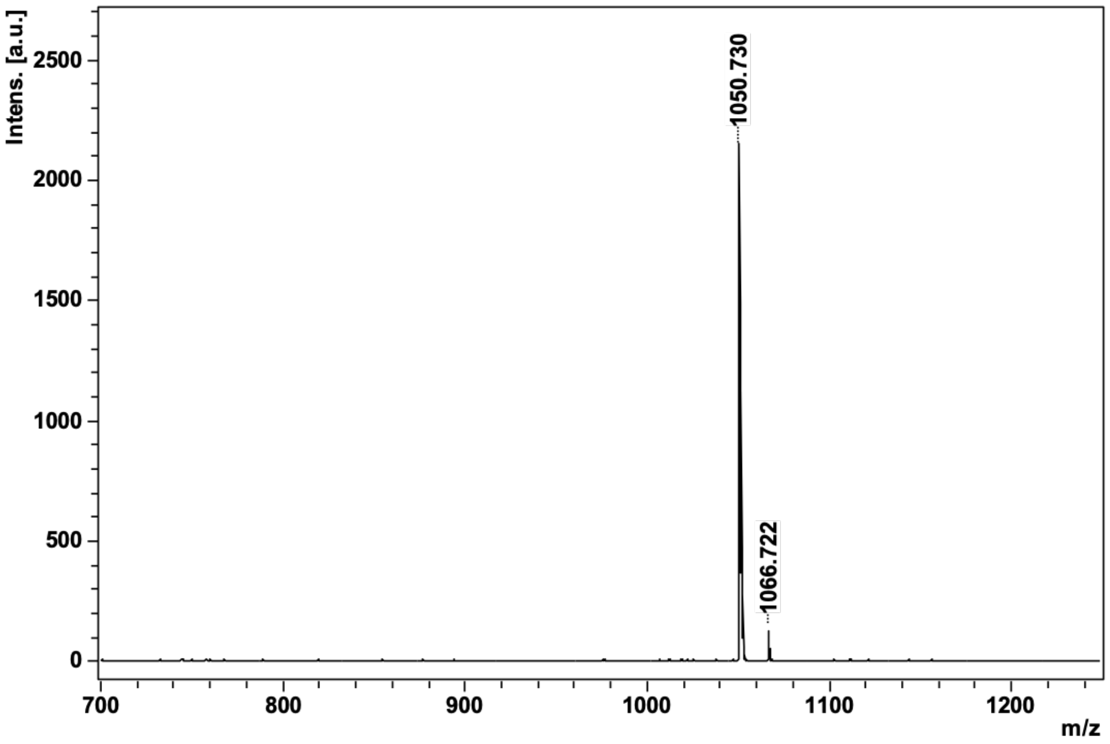

Figure S28. MALDI-TOF MS analysis of LSP6. HRMS calculated for $C_{54}H_{61}N_9O_{12}Na$ $[M+Na]^+$ 1050.433; found 1050.730. $C_{54}H_{61}N_9O_{12}K$ $[M+K]^+$ 1066.407; found 1066.722.

LSP-7

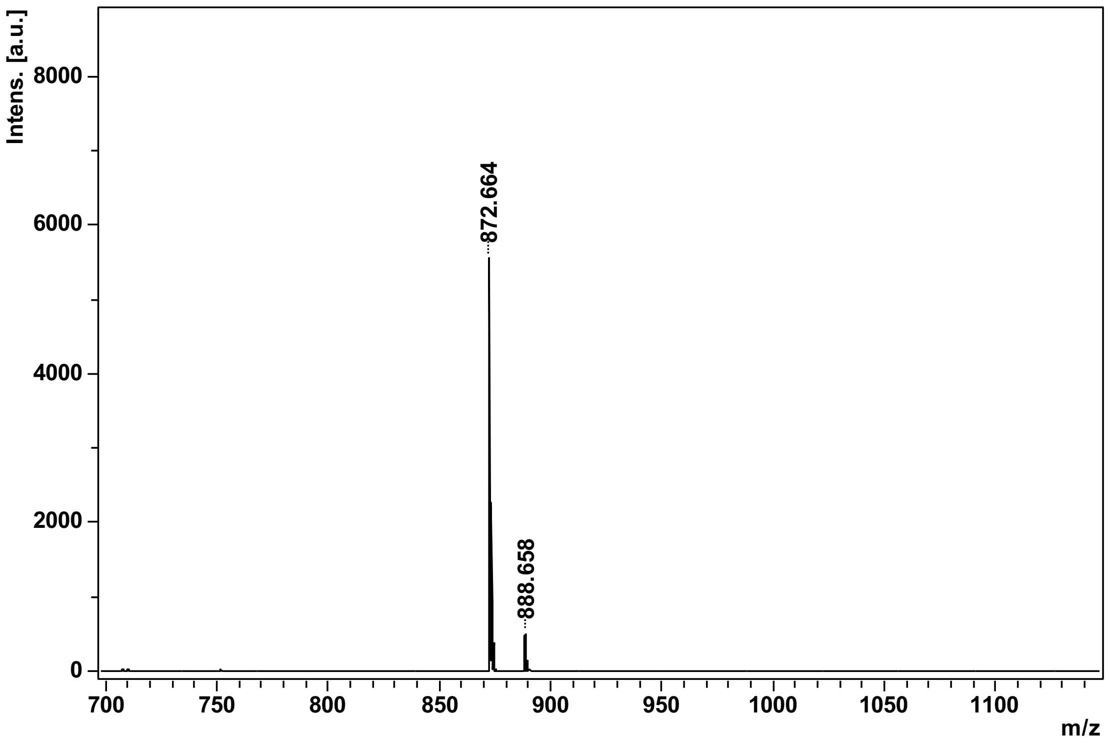

Figure S29. MALDI-TOF MS analysis of LSP7. HRMS calculated for $C_{43}H_{47}N_9O_{10}Na$ $[M+Na]^+$ 872.334; found 872.664. $C_{43}H_{47}N_9O_{10}K$ $[M+K]^+$ 888.308; found 888.658.

PROTAC-DBCO

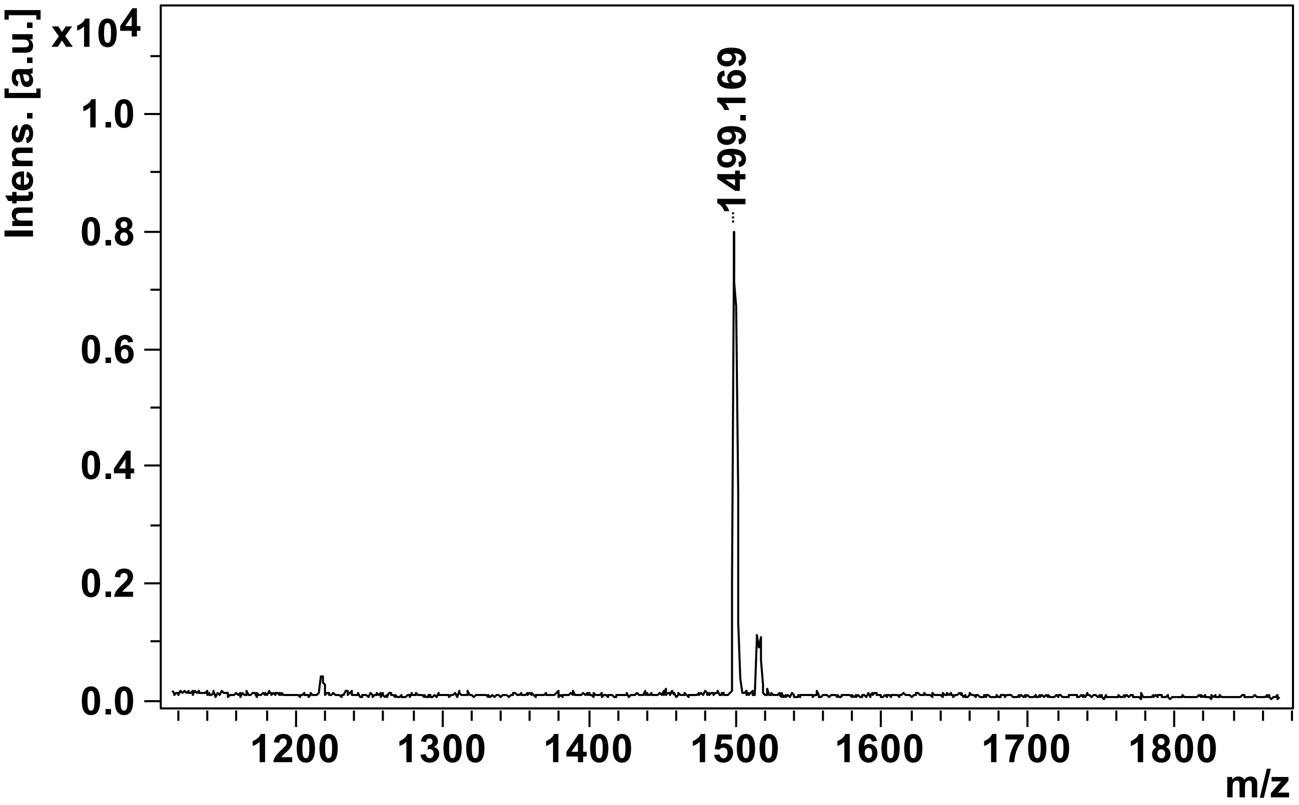

Figure S30. MALDI-TOF MS analysis of PROTAC-DBCO. HRMS calculated for $C_{76}H_{89}ClN_{12}O_{13}S_2Na$ $[M+Na]^+$ 1499.569; found 1499.169.

REFERENCE

(1) Ho, P.-Y.; Chou, T. Y.; Kam, C.; Huang, W.; He, Z.; Ngan, A. H. W; Chen, S. A Dual Organelle-Targeting Mechanosensitive Probe. *Sci. Adv.* **2023**, *9*, eabn5390.

(2) Ye, S.; Latham, A. P.; Tang, Y.; Hsiung, C.-H.; Chen, J.; Luo, F.; Liu, Y.; Zhang, B.; Zhang, X. Micropolarity Governs the Structural Organization of Biomolecular Condensates. *Nat. Chem. Biol.* **2024**, *20*, 443–451.
